# Rare SNP in the *HELB* gene interferes with RPA interaction and cellular function of HELB

**DOI:** 10.1093/narmme/ugaf019

**Published:** 2025-05-23

**Authors:** Bertha Osei, Benjamin H May, Joseph S Beard, Matthew D Thompson, Duah Alkam, Maroof Khan Zafar, Erik Bergstrom, Stephanie D Byrum, Eric J Enemark, Kirk L West, Alicia K Byrd

**Affiliations:** Department of Biochemistry and Molecular Biology, University of Arkansas for Medical Sciences, Little Rock, AR 72205, United States; Department of Biochemistry and Molecular Biology, University of Arkansas for Medical Sciences, Little Rock, AR 72205, United States; Department of Biochemistry and Molecular Biology, University of Arkansas for Medical Sciences, Little Rock, AR 72205, United States; Department of Biochemistry and Molecular Biology, University of Arkansas for Medical Sciences, Little Rock, AR 72205, United States; Department of Biochemistry and Molecular Biology, University of Arkansas for Medical Sciences, Little Rock, AR 72205, United States; Department of Biochemistry and Molecular Biology, University of Arkansas for Medical Sciences, Little Rock, AR 72205, United States; Department of Biological Sciences, Missouri University of Science and Technology, Rolla, MO 65401, United States; Department of Biochemistry and Molecular Biology, University of Arkansas for Medical Sciences, Little Rock, AR 72205, United States; Winthrop P. Rockefeller Cancer Institute, Little Rock, AR 72205, United States; Department of Biochemistry and Molecular Biology, University of Arkansas for Medical Sciences, Little Rock, AR 72205, United States; Winthrop P. Rockefeller Cancer Institute, Little Rock, AR 72205, United States; Department of Biochemistry and Molecular Biology, University of Arkansas for Medical Sciences, Little Rock, AR 72205, United States; Winthrop P. Rockefeller Cancer Institute, Little Rock, AR 72205, United States; Department of Biochemistry and Molecular Biology, University of Arkansas for Medical Sciences, Little Rock, AR 72205, United States; Winthrop P. Rockefeller Cancer Institute, Little Rock, AR 72205, United States

## Abstract

HELB is a human helicase involved in initiation of DNA replication, the replication stress response, and regulation of double-strand DNA break repair. rs75770066 is a low-frequency single-nucleotide polymorphism (SNP) in the *HELB* gene that affects age at natural menopause (ANM). rs75770066 results in a D506G substitution in a HELB-specific motif in the 1A domain of the helicase that contains amino acids known to interact with RPA. We found that this amino acid change has no effect on the enzymatic activity of HELB on naked DNA substrates but reduces the rate of unwinding by HELB on RPA coated substrates, likely because D506G substitution in HELB reduces interaction with RPA. This impaired interaction of D506G HELB with RPA dramatically impairs the cellular function of HELB and likely results in the effects of rs75770066 as this reduces recruitment of HELB to sites of DNA damage. Reduced recruitment of D506G–HELB to double-strand DNA breaks and the concomitant increase in homologous recombination likely alters the levels of meiotic recombination, which affects the viability of gametes. Because menopause occurs when oocyte levels drop below a minimum threshold, altered repair of meiotic double-stranded DNA breaks has the potential to directly affect the ANM.

## Graphical Abstract

**Graphical Abstract:**
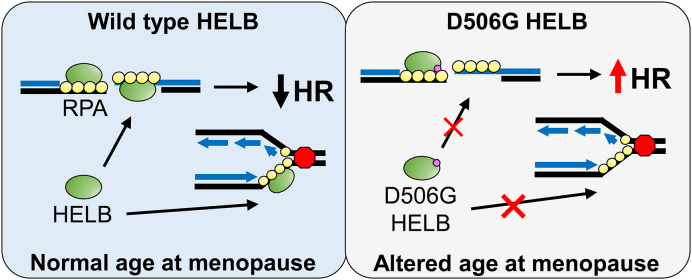

## Introduction

Menopause is a normal part of aging in women. However, in addition to the loss of fertility, the decrease in estrogen levels after menopause leads to changes in disease risk in postmenopausal women [1]. Menopause is a specific point in time 12 months after a woman’s last period, but the menopause transition, during which the levels of estrogen and progesterone produced by the ovaries vary widely, lasts for ∼7 years before menopause [2]. Incidence of cardiovascular disease increases two-fold after menopause [3, 4] and bone mineral density, which is inversely related to fracture risk, decreases ∼10% during the menopause transition and ∼1% annually in postmenopausal women [5, 6]. On the other hand, risks of some cancers increase in women with later menopause, with women in the highest quintile of age at natural menopause (ANM) (menopause after age 55 [7]) having a 15% increased incidence of estrogen receptor-positive breast cancer and a 10% increase in estrogen receptor-negative breast cancer [8]. Additionally, the risks of uterine and ovarian cancer increase by 42% in women who enter menopause after age 55 [9].

Because menopause occurs when the oocyte pool is depleted to the point that it is insufficient to support ovulation [10, 11], factors affecting the size and quality of the oocyte pool are directly related to ANM. Recent genome-wide association studies (GWAS) have illustrated that factors affecting genome stability play critical roles in regulation of reproductive senescence [8, 12–14]. Two-thirds of the genomic regions containing ANM associated single-nucleotide polymorphisms (SNPs) are DNA damage response genes [8, 12].

Multiple SNPs in the gene encoding the HELB helicase have been independently linked to ANM [8, 14–16]. Of particular interest is rs75770066, a low-frequency, nonsynonymous SNP in *HELB*. Multiple reports link rs75770066 to an increase in ANM, but a decrease in ANM has also been reported [8, 14–16]. These conflicting results are explained by a heterozygote effect—a single copy of rs75770066 increases ANM by ∼1 year while women homozygous for the rare allele have an ANM ∼1 year earlier [14]. Because rs75770066 is a low-frequency variant (minor allele frequency = 3.6%), <0.2% of women are homozygous for the rare allele, while 6.9% of women are heterozygous.

Because multiple SNPs in HELB are independently associated with ANM, this suggests that HELB is a critical component of a process affecting reproductive senescence, but the mechanism of this relationship is unclear. HELB is a superfamily 1B (SF1B) DNA helicase conserved in vertebrates [17, 18] that unwinds DNA with a 5′-to-3′ polarity [19]. HELB is important for timely entry into S phase [19, 20], possibly by promoting formation of the pre-initiation complex [20]. HELB also enhances survival of cells from DNA replication stress [21], and regulates homologous recombination (HR) for repair of double-stranded DNA breaks (DSBs) [22, 23]. HELB interacts with the single-stranded DNA (ssDNA)-binding protein, RPA [21, 23], and this interaction is essential for HELB recruitment to laser induced micro-irradiation [23]. HELB can also remove RPA filaments from ssDNA [24]. However, the relationship between these cellular functions of HELB and alterations in ANM caused by rs75770066 is unknown.

**Figure 1.**
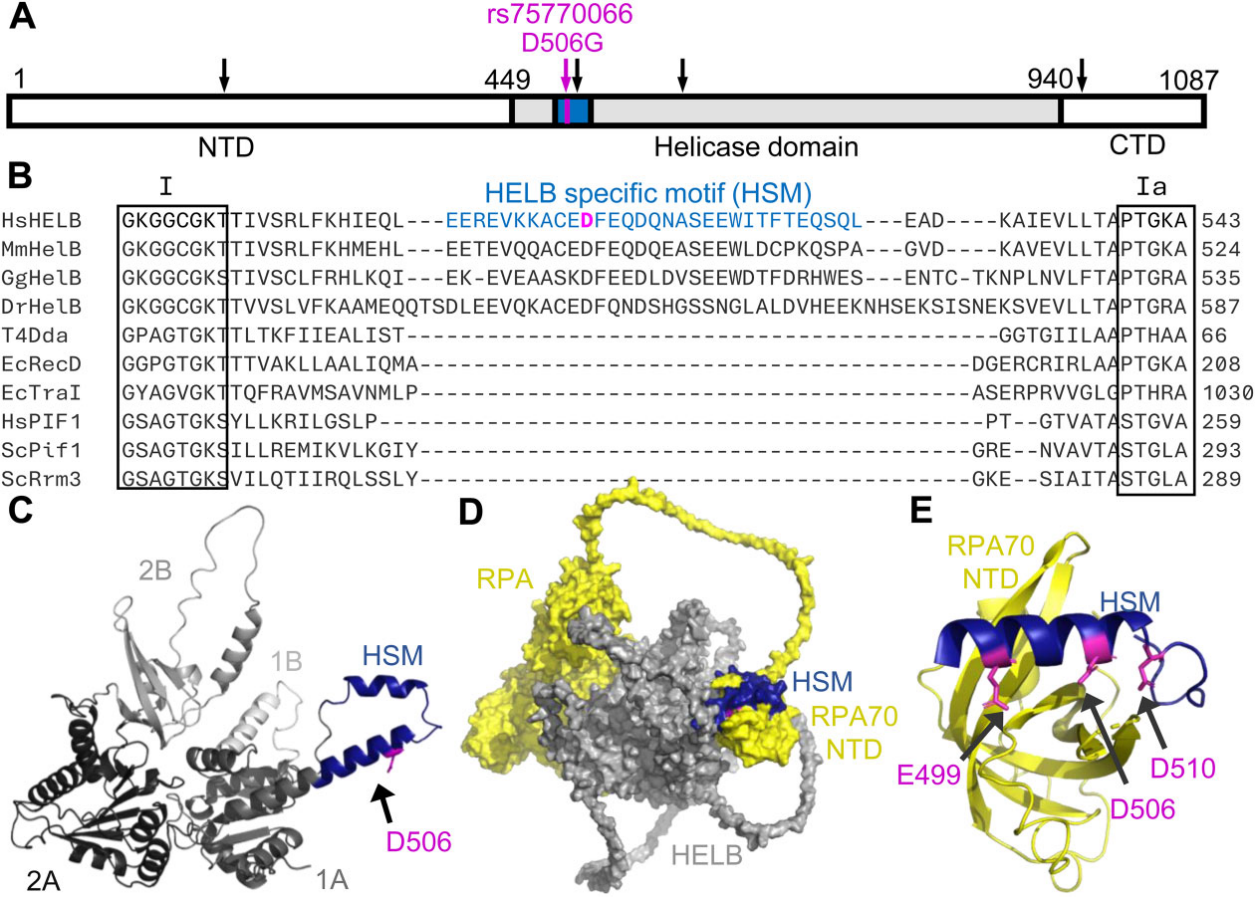
The HSM is an insertion within the 1A helicase domain that interacts with RPA. (**A**) Schematic of the HELB protein. The helicase domain is shaded with the HSM in blue. The NTD and C-terminal domain (CTD) are shown in white. Locations of amino acid substitutions due to SNPs associated with ANM are marked with arrows. (**B**) Sequence alignments of *Homo sapiens* HELB, *Mus musculus* HelB, *Gallus gallus* HelB, *Danio rerio* HelB, Bacteriophage T4 Dda, *Escherichia coli* RecD, *E. coli* TraI, *Homo sapiens* PIF1, *Saccharomyces cerevisiae* Pif1, and *Saccharomyces cerevisiae* Rrm3 helicases by Clustal Omega shows an HSM (blue) exists between helicase motifs I and Ia. D506 (magenta) is located within this HSM. (**C**) An AlphaFold structure prediction of the HELB helicase domain (gray) illustrates the HSM (blue) is an insertion within the helicase domain. The 1A and 2A conserved RecA-like domains are colored in dark gray and black, respectively. The predicted strand separation wedge (1B) is colored in light gray, and the 2B accessory domain is colored in medium gray. (**D**) An AlphaFold Multimer prediction of the complex formed between HELB (gray) and trimeric RPA (yellow) predicts interaction of the HSM with the RPA70 NTD. The apparent interactions of the RPA core with HELB (left side of D) are low confidence predictions (Supplementary Fig. S2). (**E**) A crystal structure of a HELB peptide with the RPA70 NTD (PDB: 7XV1) shows that E499, D506, and D510 (all magenta) interact with RPA [28].

HELB contains an N-terminal domain (NTD) that is involved in protein–protein interactions and may bind to DNA [20, 24], a central helicase domain, and a C-terminal subcellular localization domain [25] (Fig. 1A). rs75770066 results in a D506G substitution in HELB. D506 is located in a motif (amino acids 496–526) between helicase motifs I and Ia within the helicase domain that is conserved in HELB family members but lacking in other superfamily 1B helicases (Fig. 1A and B, and Supplementary Fig. S1) [17]. This HELB-specific motif (HSM) partially overlaps the acidic motif (amino acids 493–517) previously identified by the Fanning lab [21] and is predicted by AlphaFold to extend from the leading edge of the 1A domain of the helicase (Fig. 1C) [26, 27]. This region contains three residues that interact with RPA: E499, D506, and D510 [21]. Substitution of all three residues with alanine (E499A/D506A/D510A, hereafter 3xA) interferes with HELB interaction with RPA [21] and reduces HELB localization to sites of laser-induced microirradiation [23]. Consistent with biochemical data, AlphaFold predicts that the HSM interacts with the RPA70 NTD (Fig. 1D) [26]. A recent structure of a peptide containing a portion of the HSM containing E499, D506, and D510, and the RPA70 NTD confirms the interaction of these residues with RPA (Fig. 1E) [28]. To gain insight into the mechanism by which rs75770066 alters ANM, we measured the effect of a D506G substitution in HELB on helicase activity, protein–protein interactions, and the cellular activities of HELB.

## Materials and methods

### AlphaFold prediction

The amino acid sequences for HELB (NP_001357214.1), RPA14 (NP_002938.1), RPA32 (NP_002937.1), and RPA70 (NP_002936.1) were supplied to AlphaFold-Multimer, version 2.3.1 [26, 29], one copy of each sequence. The program was setup with default parameters, which generates five models with five predictions each for a total of 25 multimer structure predictions. The database date cutoff was set to 2023-01-10. The highest-ranking model had a scores of ptm: 0.522, iptm: 0.451, and rank: 0.465. The predicted aligned error generated by AlphaFold for this model was plotted with ChimeraX [30–32] (Supplementary Fig. S2), and its PDB coordinate file was evaluated and illustrated with PyMOL [33].

### Plasmids and oligonucleotides

A plasmid encoding human HELB, codon optimized for expression in *Escherichia coli* was ordered from GenScript and subcloned into the pSUMO vector (Life Sensors). After cleavage of the 6xHis-SUMO tag from the recombinant protein, the sequence is identical to Q8NG08. pGro7 plasmid (Takara) encodes *E. coli* groES and groEL. The pET24d-Ulp1 plasmid encoding the catalytic domain (amino acids 403–621) of *Saccharomyces cerevisiae* Ulp1 with a C-terminal His-tag was a gift from Craig Cameron [34]. The pcDNA5/FRT/TO-GFP-HELB plasmid for expression of siRNA resistant GFP-tagged HELB in human cells was a gift from Daniel Durocher [23]. The pcDNA5/FRT/TO-SFB-HELB plasmid was created by replacing the GFP sequence with an S-tag, 2X Flag tag, and streptavidin-binding peptide (SFB) [35] by Gibson Assembly. pcDNA5/FRT/TO plasmids encoding D506G HELB and E499A/D506A/D510A (3xA) HELB were created using site-directed mutagenesis. A plasmid expressing the tag only, pcDNA5/FRT/TO-SFB-EV (empty vector), was generated by Gibson Assembly. pSpCas9(BB)-2A-Puro (PX459) was a gift from Feng Zhang (Addgene plasmid #62988) [36]. p11d-tRPA(123) was a gift from Marc Wold (Addgene plasmid #102613) [37]. pSUMO-RPA70 NTD encoding amino acids 1–120 of RPA70 was created by HiFi DNA assembly using p11d-tRPA (123) and pSUMO. pCBASceI was a gift from Maria Jasin (Addgene plasmid #26477) [38].

Oligonucleotides were ordered from Integrated DNA Technologies; sequences are listed in Supplementary Table S1. siGENOME small interfering RNAs (siRNAs) were ordered from Horizon Discovery. siGENOME targeting human HELB (D-013541-02: ACAGUCGAACGUUACUUUC) and siGENOME nontargeting siRNA Pool #1 (D-001206-13) were used.

### Cell culture

HEK293T (ATCC CRL-3216), U2OS (ATCC HTB-96), U2OS-265 DSB reporter [39], and U2OS DR-GFP (ATCC CRL-3455) cells were grown in Dulbecco's Modified Eagle Medium (DMEM) (Gibco) supplemented with 10% FBS (R&D solutions S11150) at 37°C with 5% CO_2_. U2OS-265 DSB reporter cells were a generous gift from Roger Greenberg [39].

### HELB expression and purification

pSUMO-HELB and pGro7 plasmids were co-transformed into BL21(DE3) *E. coli*. Cultures were grown in NZCYM media containing 30 μg/ml kanamycin, 20 μg/ml chloramphenicol, and 0.5 mg/ml of L-arabinose to an OD_600_ of ∼1.0. Protein expression was induced by the addition of 0.1 mM isopropyl β-d-1-thiogalactopyranoside (IPTG). After growth overnight at 15°C, 250 rpm, cultures were harvested and cell pellets were resuspended in lysis buffer with protease inhibitors [50 mM HEPES, pH 8.0, 20 mM imidazole, 300 mM NaCl, 5 mM β-mercaptoethanol, 10% glycerol, Pierce EDTA-free protease inhibitor tablets, and 4 mM phenylmethylsulfonyl fluoride (PMSF)]. Cells were lysed by passing through a Microfluidizer at 18 000 psi. After centrifugation at 4°C for 90 min at 48 400 × *g*, the cleared lysate was filtered and loaded onto a Nuvia IMAC column (Bio-Rad) and eluted with 50 mM HEPES, pH 8.0, 150 mM imidazole, 300 mM NaCl, 5 mM β-mercaptoethanol, and 10% glycerol. Fractions containing His-SUMO-HELB were pooled and the His-SUMO tag was cleaved with Ulp1 during dialysis against lysis buffer lacking protease inhibitors before reloading onto the Nuvia IMAC column. The flow through containing HELB was collected and dialyzed to an intermediate salt buffer (50 mM HEPES, pH 8.0, 150 mM NaCl, 5 mM β-mercaptoethanol, and 10% glycerol) and concentrated to ∼2 ml before loading onto a GE Healthcare HiLoad 16/600 Superdex 200 pg in intermediate salt buffer. Fractions containing HELB were dialyzed to low salt buffer (50 mM HEPES, pH 8.0, 90 mM NaCl, 5 mM β-mercaptoethanol, and 10% glycerol). Protein was loaded onto a HiTrap Heparin HP column (GE Healthcare) in low salt buffer and eluted with a 16-column volume gradient from low salt to high salt (50 mM HEPES, pH 8.0, 1 M NaCl, 5 mM β-mercaptoethanol, and 10% glycerol). Fractions containing HELB were concentrated and dialyzed to an intermediate salt storage buffer (25 mM HEPES, pH 8.0, 200 mM NaCl, 5 mM β-mercaptoethanol, 1 mM EDTA, and 20% glycerol) before dialyzing to storage buffer (25 mM HEPES, pH 8.0, 100 mM NaCl, 5 mM β-mercaptoethanol, 1 mM EDTA, and 20% glycerol) for storage at −80°C. A Coomassie Plus Protein assay kit (Pierce) with bovine serum albumin (BSA) as a standard was used to determine the protein concentration, and the identity of the protein was confirmed by western blotting.

### Ulp1 expression and purification

Ulp1 was purified as described [40]. Briefly, pET24d-Ulp1 was transformed into Rosetta2(DE3) cells, and cells were grown to an OD_600_ of 0.9 before induction of protein expression by addition of 1 mM IPTG. After growth overnight at 18°C, 250 rpm, cultures were harvested and cell pellets were resuspended in lysis buffer (50 mM sodium phosphate, pH 7.5, 300 mM NaCl, 5 mM β-mercaptoethanol, and 10% glycerol) with EDTA-free protease inhibitor tablets (Pierce). Cells were lysed by passing through a Microfluidizer at 18 000 psi. After centrifugation at 4°C for 1 h at 174 000 × *g*, the cleared lysate was loaded onto a Nuvia IMAC Ni-Charged column (Bio-Rad), and proteins were eluted with 50 mM sodium phosphate, pH 7.5, 300 mM NaCl, 300 mM imidazole, 5 mM β-mercaptoethanol, and 10% glycerol. Fractions containing Ulp1 were pooled, and the buffer was exchanged to 50 mM sodium phosphate, pH 7.5, 300 mM NaCl, 5 mM β-mercaptoethanol, and 20% glycerol for storage. A Coomassie Plus Protein assay kit (Pierce) with BSA as a standard was used to determine the protein concentration.

### RPA70 NTD expression and purification

pSUMO-RPA70 NTD plasmid was transformed into BL21(DE3) *E. coli*. Cultures were grown in LB media containing 30 μg/ml kanamycin to an OD_600_ of ∼0.7. Protein expression was induced by addition of 0.5 mM IPTG. Following growth overnight at 20°C, 250 rpm, cultures were harvested, and cell pellets were resuspended in lysis buffer with protease inhibitors (25 mM HEPES, pH 8.0, 20 mM imidazole, 200 mM NaCl, 5 mM β-mercaptoethanol, 10% glycerol, and Pierce EDTA-free protease inhibitor tablets). Cells were lysed by passing through a Microfluidizer at 18 000 psi. After centrifugation at 4°C for 120 min at 48 400 × *g*, the cleared lysate was filtered and loaded onto a Nuvia IMAC column (Bio-Rad), washed with a high salt wash buffer (25 mM HEPES pH 8.0, 20 mM imidazole, 1 M NaCl, 10% glycerol, and 5 mM β-mercaptoethanol), an ATP wash containing 2 mM ATP and 3 mM MgCl_2_ in lysis buffer lacking protease inhibitors, and eluted with 25 mM HEPES, pH 8.0, 200 mM imidazole, 200 mM NaCl, 5 mM β-mercaptoethanol, and 10% glycerol. Fractions that contained the His-SUMO-RPA70 NTD were pooled, and the His-SUMO tag was cleaved with Ulp1 during dialysis against lysis buffer lacking protease inhibitors before reloading onto the Nuvia IMAC column. The flow through containing RPA70 NTD was collected and dialyzed to an intermediate salt buffer (50 mM HEPES pH 8.0, 150 mM NaCl, 0.5 mM EDTA, 5 mM β-mercaptoethanol, and 10% glycerol) and concentrated to ∼2 ml before loading onto a GE Healthcare HiLoad 16/600 Superdex 200 pg in intermediate salt buffer. Fractions containing RPA70 NTD were dialyzed into a storage buffer (25 mM HEPES, pH 8.0, 150 mM NaCl, 2 mM β-mercaptoethanol, 1 mM EDTA, and 20% glycerol) for storage at −80°C. A Coomassie Plus Protein assay kit (Pierce) with BSA as a standard was used to determine the protein concentration.

### RPA expression and purification

Heterotrimeric RPA was purified as described [41]. Briefly, p11d-tRPA(123) expressing RPA70, RPA32, and RPA14 was transformed into BL21(DE3) *E. coli*. Cultures were grown in Terrific Broth containing 100 μg/ml ampicillin with a single colony from a day-old terrific agar plate. Cell cultures were grown at 37°C overnight without aeration to an OD_600_ of 0.5–0.8. Protein expression was induced by addition of 0.3 mM IPTG. Following growth for 2–3 h at 37°C, 250 rpm, cultures were harvested, and cell pellets were resuspended in HI buffer [30 mM HEPES, pH 7.8, 0.25 M EDTA, 0.25% myo-inositol, 1 mM dithiothreitol, 0.01% NP-40 (IGEPAL CA-630)] with protease inhibitors (1 mM PMSF and Pierce EDTA-free protease inhibitor tablets). Cells were lysed by passing through a Microfluidizer at 10 000 psi. After centrifugation at 4°C for 35 min at 23 708 × *g*, the cleared lysate was filtered and loaded onto an Affi-Gel Blue column (Bio-Rad), washed with a HI buffer with 50 mM KCl, HI buffer with 800 mM KCl, and HI buffer with 500 mM NaSCN. RPA was eluted with HI buffer containing 1.5 M NaSCN and immediately loaded onto a CHT XT column (Bio-Rad) equilibrated in HI buffer with 30 mM KCl. The column was washed with HI buffer, and RPA was eluted in 80 mM potassium phosphate, pH 7.5, 0.25 M EDTA, 0.25% myo-inositol, 1 mM dithiothreitol, and 0.01% NP-40. The entire 80 mM potassium phosphate eluate was loaded onto a High-Q column (Bio-Rad) that was equilibrated in HI buffer with 50 mM KCl. The column was washed with HI buffer with 50 mM KCl followed by HI buffer with 100 mM KCl. RPA was eluted in HI buffer with a gradient of KCl from 200 to 400 mM. Fractions containing RPA were stored at −80°C, and the concentration was determined using a Coomassie Plus Protein assay kit (Pierce) with BSA as a standard. The identity of the proteins was confirmed by western blotting.

### DNA binding

Serial dilutions of HELB in assay buffer (25 mM HEPES pH 7.5, 10 mM KOAc, 10 mM Mg(OAc)_2_, 0.1 mM EDTA, 2 mM β-mercaptoethanol, and 0.1 mg/ml BSA) were mixed with 1 nM 3′-6-carboxyfluorescein (FAM)-labeled T_16_ and incubated at room temperature for 30 min before measuring polarization in a VICTOR Nivo Multimode Plate Reader (Perkin Elmer) at room temperature with excitation through a 480/30 nm bandpass filter and emission after a 530/30 nm bandpass filter. The total fluorescence intensity was also measured following the 30-min incubation to ensure that the fluorescence intensity remained constant throughout the experiment. Polarization was converted to anisotropy, and data were fit using the quadratic equation to obtain the equilibrium dissociation constants.

### DNA unwinding

A 5′-FAM labeled 30 base pair forked duplex with a 30-nucleotide loading site on the 5′-end and a 10-nucleotide overhang on the 3′-end (sequences are in Supplementary Table S1) was prepared by mixing at a 1:1.2 ratio of fluorescently labeled to unlabeled strand and heating at 95°C for 10 min followed by slowly cooling to room temperature. For unwinding with RPA, the substrate contained a 70-nucleotide loading site on the 5′-end and a 10-nucleotide overhang on the 3′-end. Substrate (20 nM) was incubated with 200 nM HELB in assay buffer (25 mM Hepes pH 7.5, 10 mM KOAc, 0.1 mM EDTA, 2 mM β-mercaptoethanol, and 0.1 mg/ml BSA) for 5 min before initiating the reaction at 25°C by addition of 2.5 mM ATP and 5 mM Mg(OAc)_2_. The concentrations given are final, after mixing. An annealing trap (100 nM) complementary to the unlabeled strand was added with the ATP to prevent reannealing of the product strands. For unwinding with RPA, 20 nM substrate was incubated with 75 nM RPA, 2.5 mM ATP, and 5 mM Mg(OAc)_2_ in assay buffer for 5 min before initiating the reaction at 25°C by addition of 100 nM annealing trap and 200 mM HELB. Samples were removed at various times and quenched by addition of an equal volume of 400 mM EDTA, 300 nM annealing trap, 2 μM T_50_ protein trap, 6% glycerol, and 0.1% Orange G. Samples were separated by native 20% PAGE (19:1 acrylamide to bis-acrylamide) and visualized on an Amersham Typhoon RGB Imager (Cytiva) with a 488 nm laser and 525BP20 emission filter. The quantity of double-stranded DNA (dsDNA) and ssDNA at each time point was quantified using ImageQuantTL (Cytiva), and data were fit to a single exponential function to determine the rate constants for unwinding.

### Generation of HELB knockout cells

CRISPR–Cas9 was used to generate HELB knockout clones in U2OS and HEK293T cell lines as described previously [36]. Briefly, two guide RNAs (gRNAs) targeting exon 1 in all splice variants of HELB were generated using the online tool at www.atum.bio/eCommerce/cas9/input and were synthesized by Integrated DNA Technologies. The sequences are listed in Supplementary Table S2. The gRNAs were annealed, phosphorylated by T4 polynucleotide kinase, and ligated into the BsbI digested pSpCas9(BB)-2A-Puro (PX459) plasmid with T7 DNA ligase. Next, exonuclease digestion of residual linear plasmid was conducted by PlasmidSafe Exonuclease. Cells were then transfected with the construct encoding the appropriate gRNA. pSpCas9(HELB gRNA) plasmid (10 μg) was mixed with 50 μl of 1 mg/ml polyethylenimine (PEI, Polysciences, Inc) in 37°C OptiMEM by vortexing. Following a 30 min incubation at room temperature, plasmid mix was added dropwise to cells cultured in antibiotic-free DMEM. After 24 h of transfection, clones were selected for 48 h with 2 μg/ml of puromycin prior to plating and isolating individual clones. The HELB knockout clones were validated by western blotting (Supplementary Fig. S3A and B).

### Western blotting

Cells were harvested and resuspended in lysis buffer [40 mM HEPES pH 7.5, 10 mM NaCl, 1% Triton X-100, 1× protease inhibitor cocktail (Sigma P2714), 20 mM β-glycerophosphate, 1 mM Na_2_VO_4,_ and 1 mM dithiothreitol]. Proteins were separated by 7.5% SDS–PAGE, and the proteins were transferred to a 0.45 μm nitrocellulose membrane. The membrane was blocked overnight with 5% nonfat milk in 1× TBS with 0.1% Tween-20 (TBS-T). Membranes were incubated with primary antibodies in TBS-T with 1% milk for 1 h at room temperature. Then membranes were incubated with fluorescent or HRP-conjugated secondary antibodies in TBS-T with 1% milk for 1 h at room temperature. HRP blots were imaged using Amersham ECL Prime (GE Healthcare) while fluorescent blots were directly imaged on a ChemiDoc MP imaging system (Bio-Rad). Antibodies used were rabbit anti-HELB (Abcam: ab202141, 1:10000–1:5000), rabbit anti-RPA70 (Cell Signaling 2267S, 1:1000), mouse anti-β-actin (Cell signaling: 3700S, 1:2000) or hFAB rhodamine anti-β-actin (Bio-Rad: 10000068189 C, 1:5000), mouse anti-FLAG (Sigma F1804, 1:1000), mouse anti-H4 (Cell Signaling: L64C1, 1:1000), anti-GAPDH hFAB rhodamine (Bio-Rad: 12004168, 1:5000), HRP-labeled goat anti-mouse IgG (Perkin Elmer: NEF822001EA, 1:10000), HRP-labeled goat anti-rabbit IgG (Perkin Elmer: NEF812001EA, 1:10000), StarBright Blue 520 goat anti-mouse IgG (Bio-Rad 64456855, 1:2500), and StarBright Blue 700 goat anti-rabbit (Bio-Rad 64484700, 1:2500).

### Tandem affinity purification mass spectrometry

HELB^KO^ HEK293T cells were transfected with plasmids encoding pcDNA5/FRT/TO-SFB-HELB variants or pcDNA5/FRT/TO-SFB-EV using PEI (Polysciences, Inc). After 24 h, the cells were expanded, and 48 h after transfection, the cells were harvested by trypsinization. The cell pellet was lysed in NETN [150 mM NaCl, 0.5 mM EDTA, 20 mM Tris–Cl pH 8.0, 0.5% NP-40 Alternative (Millipore 492016)] supplemented with 1 μg/ml aprotinin, and 1 μg/ml pepstatin A with rotation for 1 h at 4°C. An aliquot of the lysate was removed and saved to evaluate proteins that were enriched from the lysate. The samples were centrifuged at 9000 × *g* for 20 min at 4°C. The supernatant (soluble fraction) was transferred to a new tube. Streptavidin Sepharose High Performance (Cytiva) was added to the tubes containing the supernatants, and the samples were rotated for 1 h at 4°C.

While the soluble fraction was rotating, the chromatin pellet was washed with phosphate buffered saline (PBS) before adding NETN supplemented with 1 μg/ml aprotinin, 1 μg/ml pepstatin A, 10 mM MgCl_2_, and 200 U Turbonuclease from *Serratia marcescens* (Sigma). Samples were rotated for 1 h at 4°C before centrifuging at 9000 × *g* for 20 min at 4°C. The supernatants (chromatin fraction) were transferred to new tubes. Streptavidin Sepharose High Performance (Cytiva) was added to the tubes containing the supernatants, and the samples were rotated for 1 h at 4°C.

Soluble and chromatin samples were centrifuged at 211 × *g* for 2 min before aspirating the supernatant. The pellets were resuspended in NETN and transferred to a clean tube where the pellets were washed three times with NETN. After aspiration of the last wash, SFB-tagged protein complexes were eluted by addition of 2 mg/ml biotin in NETN with rotation for 1 h at 4°C followed by centrifugation at 211*× g* for 1 min. The supernatant was transferred to a new tube and the biotin elution was repeated. After the second biotin elution, S protein agarose (Millipore) was added to each sample, and the samples were rotated for 1 h at 4°C before washing three times with NETN.

To remove detergents and salts for mass spectrometry, the beads were washed in freshly prepared, cold, 100 mM ammonium bicarbonate, transferred to a new tube, and washed a second time. After removing the supernatant, the beads were frozen at −80°C and submitted to the IDeA National Resource for Quantitative Proteomics for analysis. Experiments were performed in biological quintuplicate.

Protein samples were reduced, alkylated, and digested using filter-aided sample preparation (FASP) [42] with sequencing grade modified porcine trypsin (Promega). Tryptic peptides were then separated by reverse phase XSelect CSH C18 2.5 μm resin (Waters) on an in-line 150 × 0.075 mm column using an UltiMate 3000 RSLCnano system (ThermoScientific). Peptides were eluted using a 60 min gradient from 98:2 to 65:35 buffer A:B ratio (buffer A is 0.1% formic acid, 0.5% acetonitrile and buffer B is 0.1% formic acid, 99.9% acetonitrile). Eluted peptides were ionized by electrospray (2.4 kV) followed by mass spectrometric analysis on an Orbitrap Eclipse Tribrid mass spectrometer (ThermoScientific) configured to acquire a precursor scan (385–1015 *m/z*, 60 000 resolution, normalized AGC target 100%, maximum injection time 50 ms) followed by 50 × 12 m/z DIA spectra (12 *m/z* precursor isolation windows at 15 000 resolution, normalized AGC target 100%, maximum injection time 33 ms) using a staggered window pattern with optimized window placements. Precursor spectra were acquired after each DIA duty cycle.

Following data acquisition, data were searched using Spectronaut (Biognosys version 18.5) against the UniProt *Homo sapiens* database (April 2023) using the directDIA method with an identification precursor and protein *q*-value cutoff of 1%, generate decoys set to true, the protein inference workflow set to maxLFQ, inference algorithm set to IDPicker, quantity level set to MS2, cross-run normalization set to false, and the protein grouping quantification set to median peptide and precursor quantity. Protein MS2 intensity values were assessed for quality using ProteiNorm [43]. The data were normalized using variance stabilizing normalization (VSN) [44] and analyzed using R package proteoDA to perform statistical analysis using Linear Models for Microarray Data (limma) with empirical Bayes (eBayes) smoothing to the standard errors [45, 46]. Each lysate sample was compared with the TAP sample in order to evaluate the proteins enriched in the TAP compared to the initial protein amounts. Separately, the treatment conditions for each TAP sample were compared. Proteins with an FDR adjusted *P*-value < 0.05 and a fold change >2 were considered significant.

### Streptavidin affinity purification

HELB^KO^ HEK293T cells were transfected with plasmids encoding pcDNA5/FRT/TO-SFB-HELB variants using PEI (Polysciences, Inc). After 24 h, cells were harvested by trypsinization. The cell pellet was lysed in NETN [150 mM NaCl, 0.5 mM EDTA, 20 mM Tris–Cl pH 8.0, 0.5% NP-40 Alternative (Millipore # 492016)] supplemented with 1 μg/ml aprotinin, 2 μg/ml pepstatin A, 10 mM MgCl_2_, and 100 U Turbonuclease from *Serratia marcescens* (Sigma). Samples were rotated for 1 h at 4°C. Samples were centrifuged at 21 130 × *g* for 20 min at 4°C. The supernatants were transferred to clean tubes, and a sample of the supernatant was saved in a separate tube as the input sample. Streptavidin Sepharose High Performance (Cytiva) was added to the tubes containing the supernatants, and the samples were rotated for 1 h at 4°C. Samples were centrifuged at 211 × *g* for 1 min before aspirating the supernatant. The pellets were washed three times with NETN. After aspiration of the last wash, Laemmli buffer was added to the beads and the reserved input sample. Both were heated at 95°C for 5 min before separation by 7.5% sodium dodecyl sulfate–polyacrylamide gel electrophoresis (SDS–PAGE) and western blotting.

### Peptide binding

Serial dilutions of RPA70 NTD in assay buffer (20 mM HEPES pH 7.5, 20 mM NaCl, 0.2 mM dithiothreitol, and 0.1 mg/ml BSA) were mixed with 20 nM 3′-6-FAM labeled WT HELB and D506G HELB peptides (residues 493–519) ordered from GenScript (sequences are in Supplementary Table S3). Samples were incubated at room temperature for 10 min before measuring polarization in a VICTOR Nivo Multimode Plate Reader (Perkin Elmer) at room temperature with excitation through a 480/30 nm bandpass filter and emission after a 530/30nm bandpass filter. The total fluorescence intensity was also measured following the 30 min incubation to ensure that the fluorescence intensity remained constant throughout the experiment. Polarization was converted to anisotropy and data were fit using the quadratic equation to obtain the equilibrium dissociation constants.

### Immunofluorescence

pcDNA5/FRT/TO-SFB-HELB (WT, D506G, and 3xA) plasmids (1 μg) were transfected into 1.0 × 10^5^ HELB^KO^ U2OS cells using FuGENE HD (Promega) according to the manufacturer’s protocol followed by incubation at 37°C with 5% CO_2_ for 24 h. Expression was confirmed by western blotting (Supplementary Fig. S3C). After incubation, cells were treated 10 μM camptothecin (CPT) for 1 h at 37°C with 5% CO_2_. Post treatment, cells were washed with 1× PBS before pre-extraction with 0.5% Triton X-100 for 5 min on ice. Cells were washed again with 1× PBS and fixed with 2% paraformaldehyde for 15 min at room temperature, washed again with 1× PBS, and permeabilized with a solution consisting of 20 mM HEPES, 50 mM NaCl, 3 mM MgCl_2_, 300 mM sucrose, and 0.5% Triton X-100 for 10 min on ice before blocking overnight with 5% BSA in 1× PBS. Cells were incubated with rabbit anti-FLAG antibody (Cell Signaling: 2368S, 1:1000) for 1 h at room temperature followed by goat anti-rabbit IgG Alexa Fluor 647 (Invitrogen: A21244, 1:2500) for 1 h at room temperature. After washing, the cells were mounted with 2.5 mg/ml DABCO (1,4-diazobicyclo[2.2.2]octane) and 0.1 μg/ml DAPI (4',6-diamidino-2-phenylindole) in 25% PBS, and 75% glycerol. Cells were imaged on an Olympus FV1000 confocal microscope. The intensity of HELB in the nucleus of each cell was quantified using CellProfiler [47]. Experiments were performed in triplicate.

### Chromatin fractionation

HELB^KO^ HEK293T cells (1.0 × 10^6^) were seeded 24 h prior to transfection. Plasmids (pcDNA5/FRT/TO-SFB-HELB encoding WT, D506G, or 3xA HELB) were diluted into OptiMEM (Gibco) followed by the addition of PEI (Polysciences, Inc) and a 30 min incubation at room temperature. After swapping the medium to antibiotic-free DMEM, cells were transfected with 10 μg of plasmid. After incubation overnight, cells were swapped to DMEM containing 100 U/ml penicillin and streptomycin. Twenty-four hours after transfection, cells were treated with 10 μM CPT for 1 h at 37°C with 5% CO_2_. Cells were harvested via trypsinization, and cell pellets were washed with 1× PBS. Cell pellets were lysed in NETN buffer [150 mM NaCl, 0.5 mM EDTA, 20 mM Tris–HCl pH 8.0, 0.5% NP-40 (Sigma–Aldrich Igepal CA-630)] with 10 ng/ml aprotinin and 20 ng/ml pepstatin. Aliquots were taken for whole cell extracts (Supplementary Fig. S3D) before separation of chromatin by centrifugation at 21 130 × *g* for 20 min at 4°C. The soluble fractions (supernatants) were collected and boiled in Laemmli buffer. The remaining pellet was washed three times with ice-cold 1× PBS and centrifuged at 21 130 × *g* for 1 min between each wash to remove any remaining supernatant. Chromatin pellets were extracted with 0.2 N HCl on ice for 15 min, followed by quenching with an equal volume of ice-cold 1 M Tris–Cl, pH 7.5. Extracted chromatin pellets were boiled in Laemmli buffer, and supernatants were collected as the chromatin fractions. Samples were separated by electrophoresis and immunoblotted as described in the western blotting section.

### FokI nuclease double-strand DNA break co-localization assay

U2OS-265 cells expressing a DSB reporter were a gift from Roger Greenberg [39]. Cells were seeded in a six-well dish before transfecting with plasmids encoding GFP-HELB (WT, D506G, or 3xA). The next day, cells were moved to glass coverslips. After 24 h, cells were treated with 1 μM 4-hydroxytamoxifen (4-OHT) (Sigma) and 1 μM Shield1 (Clonetech Labs) for 4 h. Cells were fixed using 3% paraformaldehyde, permeabilized, blocked for 1 h with 5% BSA, and stained with rabbit anti-mCherry (Cell Signaling 43590S, 1:200) followed by goat anti-rabbit IgG Alexa Fluor 594 (Invitrogen: A11037, 1:500) and Hoechst 33342 (Invitrogen H3570, 1:10000) before mounting with anti-fade solution [0.02% *p*-phenylenediamine (Sigma, P6001) in 90% glycerol in PBS]. Cells were imaged on a Nikon Eclipse Ti2 confocal microscope. Experiments were performed in triplicate, and localization of GFP-HELB relative to mCherry was scored blind. Z-stacks acquired from a Zeiss LSM 880 Confocal Microscope with Airyscan were used to make 3D reconstructions, compiled into movies using NIS-Element Viewer-Imaging Software, and compressed with VideoSmaller.com.

### DR-GFP HR reporter assay

DR-GFP U2OS cells (5 × 10^5^) were transfected with 89.6 pmol of siGENOME nontargeting pool and siGENOME human HELB (Horizon Discovery) using DharmaFECT 1 (Horizon Discovery). DharmaFECT 1 and siRNA were separately diluted in OptiMEM before combining and incubating for 20 min at room temperature. After addition of the transfection agent to the cells, they were incubated at 37°C with 5% CO_2_. Twenty-four hours after siRNA transfection, the medium with transfection reagent was replaced with fresh medium. Cells were transfected again with 1.8 μg of plasmid either encoding pcDNA5/FRT/TO-SFB-HELB variants or pcDNA5/FRT/TO-SFB-EV with or without pCBA-iSceI using PEI. Plasmids were mixed with OptiMEM before addition of PEI. After incubation at room temperature for 20–30 min, the transfection reaction was added to the cells gently and incubated at 37°C with 5% CO_2_. Twenty-four hours after the second transfection, the medium with transfection reagents was replaced with fresh medium and incubated again.

Seventy-two hours after siRNA transfection, cells were harvested by trypsinization followed by mixing 2:1 with 10% formaldehyde and vortexing immediately for 2–3 s on medium speed. This was followed by incubation in 3.3% formaldehyde for 30 min. Cells were centrifuged at 150 × *g* for 5 min to pellet the cells, and the formaldehyde was removed, and cells were resuspended in 500 μl of 1× PBS. The resuspended cells were transferred into round-bottom tubes with strainer snap caps and centrifuged again at 150 × *g* for 5 min to further separate cells. Cells were analyzed for green fluorescence on a BD LSRFortessa. Cells were gated by forward scatter versus side scatter. Experiments were performed in biological triplicate. Supplementary Figure S3E shows the knockdown efficiency.

## Results

### D506G substitution does not impair HELB helicase activity

**Figure 2.**
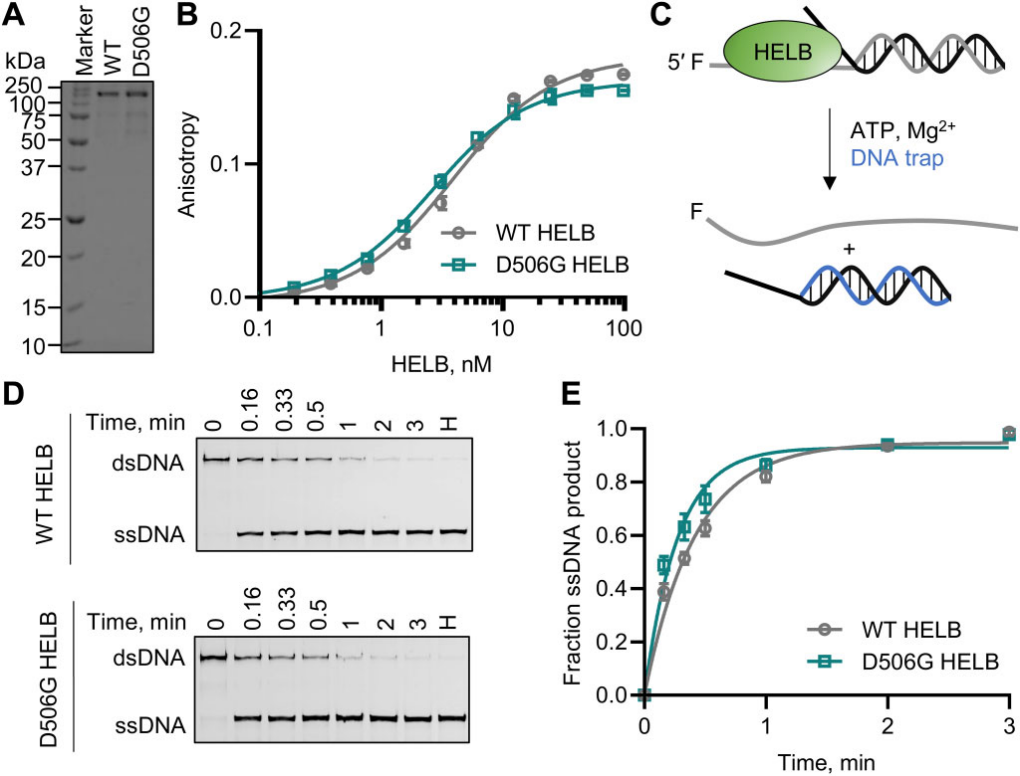
D506G substitution in HELB does not impair HELB activity. (**A**) SDS–PAGE showing purified WT and D506G HELB. (**B**) Fluorescence anisotropy of 3′-FAM-T_16_ incubated with varying concentrations of WT and D506G HELB is shown. Dissociation constants are 3.4 ± 0.2 nM and 2.2 ± 0.4 nM for binding of WT and D506G HELB, respectively. WT and D506G HELB DNA binding is not significantly different based on a paired *t*-test (*P* = 0.84). (**C**) After preincubation of HELB with a forked duplex, unwinding occurs upon addition of ATP and Mg^2+^. A DNA trap prevents reannealing of the ssDNA products. (**D**) Products were separated by native PAGE, and the data was fit to a single exponential function (**E**). Rate constants for unwinding are 2.5 ± 0.2 s^−1^ and 3.7 ± 0.5 s^−1^ for unwinding by WT HELB and D506G HELB, respectively. Data are the average and standard deviation of triplicate experiments.

To perform its function in a cell, an enzyme must be catalytically active, targeted to the appropriate location within the cell, and interact with the correct protein partners. We investigated the mechanism by which a D506G substitution in HELB interferes with the function of HELB, thereby altering the ANM, by comparing the activity of wild-type (WT) and D506G HELB that were overexpressed and purified from *E. coli* (Fig. 2A) in each of these processes. D506 is predicted (Fig. 1) to be located in an α-helix that extends from the 1A RecA domain [26, 27]. Because glycine is helix-destabilizing due to its conformational flexibility, a D506G substitution may disrupt folding of the helicase domain, which would prevent HELB from binding and unwinding DNA. We determined the effect of D506G substitution on HELB affinity for ssDNA by measuring the anisotropy of 3′-FAM-T_16_ pre-incubated with varying concentrations of WT and D506G HELB and found that the D506G HELB and WT HELB have similar affinity for ssDNA (Fig. 2B). We also compared the helicase activity of WT and D506G HELB by measuring the ability of each variant to unwind a forked partial duplex substrate (Fig. 2C). Both enzymes unwind dsDNA at similar rates (Fig. 2C–E). This indicates that D506G does not substantially alter the affinity of HELB for ssDNA or the ability of HELB to unwind duplex DNA.

### D506G substitution in HELB impairs interaction with RPA

**Figure 3.**
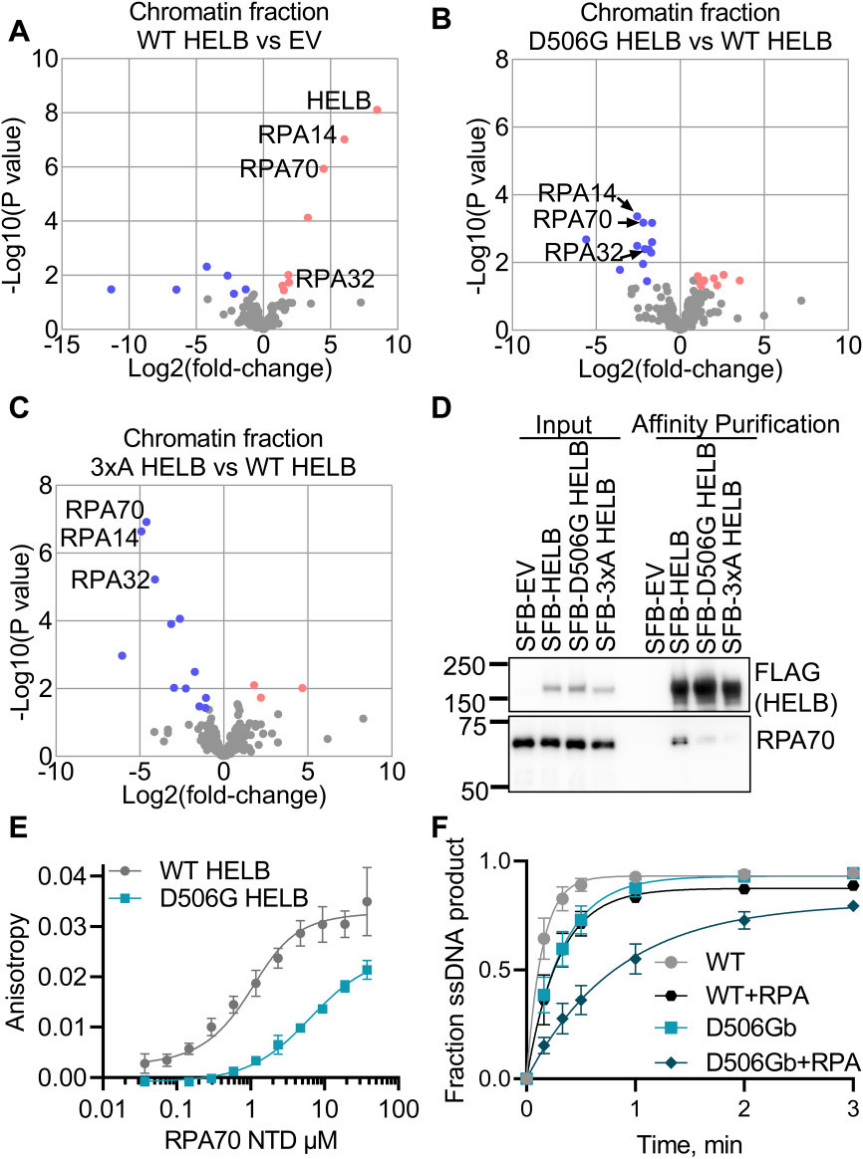
D506G substitution in HELB reduces interaction with RPA. Proteins were identified by TAP-MS from the chromatin fraction isolated from HELB^KO^ 293T cells expressing SFB-EV or SFB-HELB (WT, D506G, or 3xA) in biological quintuplicate. Significantly enriched (red) and depleted (blue) proteins are plotted for WT HELB relative to EV (**A**), D506G HELB relative to WT HELB (**B**), and 3xA HELB relative to WT HELB (**C**). (**D**) Input samples and samples after affinity purification of SFB-HELB variants on streptavidin beads were probed for FLAG and RPA70 by western blot. Additional replicates and quantification are shown in Supplementary Fig. S6. (**E**) Fluorescence anisotropy of 6-FAM labeled WT HELB or D506G HELB peptides (residues 493–519) incubated with varying concentrations of the RPA70 NTD are shown. Dissociation constants are 0.52 ± 0.16 μM and 5.9 ± 2.5 μM for binding of peptides corresponding from WT and D506G HELB, respectively. Data are the average and standard deviation of triplicate experiments. The *P*-value based on a paired *t*-test is < 0.0001. (**F**) Rate constants for unwinding of a DNA substrate with a 70T 5′-ssDNA overhang with RPA prebound to the ssDNA overhang are 3.6 ± 1.2 s^−1^ and 1.2 ± 0.3 s^−1^ for unwinding by WT HELB and D506G HELB, respectively. Rate constants for unwinding of a DNA substrate with a 70T 5′-ssDNA overhang without RPA are 7.1 ± 1.6 s^−1^ and 3.2 ± 0.7 s^−1^ for unwinding by WT HELB and D506G HELB, respectively. Data are the average and standard deviation of quadruplicate experiments. The *P*-values based on a paired repeated measures ANOVA with a Sidak’s multiple comparisons test are 0.20 for WT HELB to D506G HELB, 0.062 for WT HELB to WT HELB + RPA, 0.019 for D506G HELB to D506G HELB + RPA, and 0.033 for WT HELB + RPA to D506G HELB + RPA.

Because the D506 is one of three residues of HELB reported to interact with RPA (magenta in Fig. 1 and Supplementary Fig. S1) [21], we investigated whether a D506G substitution affects HELB interactions with other proteins. The protein interactomes of WT, D506G, and 3xA SFB-HELB in the chromatin and soluble fractions were identified using tandem affinity purification mass spectrometry (TAP-MS) (Supplementary Tables S4-S7) and compared to that of SFB-EV (Fig. 3, and Supplementary Figs S4 and S5, and Supplementary Tables S8 and S9). In the WT HELB chromatin fraction, all three subunits of RPA are highly enriched (Fig. 3A). The RPA70 and RPA14 subunits are also enriched in the D506G HELB chromatin samples (Supplementary Fig. S4A). However, the degree of enrichment is reduced in the D506G HELB samples relative to WT HELB samples, and all three subunits of RPA are depleted in the D506G HELB relative to WT HELB (Fig. 3B). This suggests that a D506G substitution in HELB interferes with interaction with RPA.

None of the subunits of RPA are significantly enriched in the 3xA HELB chromatin samples (Supplementary Fig. S4B), and they are significantly depleted in the 3xA HELB chromatin samples relative to the WT HELB chromatin samples (Fig. 3C), confirming the results from the Fanning lab that [21] that E499, D506, and D510 are critical residues for interaction with RPA. Interestingly, very few proteins are significantly enriched in the 3xA HELB chromatin samples (Supplementary Fig. S4B, and Supplementary Tables S5 and S8), suggesting that many of the HELB interacting proteins in the chromatin fraction may interact indirectly through RPA.

HELB binds to chromatin with DNA damage and replication stress [21, 23, 25] but is also present in the nucleoplasm and cytoplasm [21]. Thus, we also identified HELB interacting proteins in the soluble fraction that contains both the nucleoplasm and cytoplasm (Supplementary Fig. S5, and Supplementary Tables S6, S7, and S9). Besides HELB itself, the three subunits of RPA are the most significant highly enriched proteins in the WT HELB soluble samples (Supplementary Fig. S5A), suggesting that HELB interacts with RPA in a DNA independent manner. This is consistent with structural studies of a peptide containing a portion of the HSM including E499, D506, and D510 interacting with the RPA70 NTD in the absence of DNA [21, 28].

In the D506G HELB soluble samples, the three subunits of RPA are also enriched, although to a lesser degree than in the WT HELB soluble samples (Supplementary Fig. S5B), and all three subunits are significantly depleted in the D506G HELB soluble samples relative to the WT HELB soluble samples (Supplementary Fig. S5C). The RPA subunits are further depleted in the 3xA HELB soluble samples (Supplementary Fig. S5D), and they are significantly depleted in the 3xA HELB soluble samples relative to the WT HELB soluble samples (Supplementary Fig. S5E).

In WT HELB, D506G HELB, and 3xA HELB soluble samples, CDK2 was significantly enriched (Supplementary Fig. S5A, B, and D). CDK2 phosphorylates HELB at the G1-to-S transition [25], and this interaction with WT HELB was previously observed in cells treated with neocarzinostatin, a radiomimetic drug [23]. This interaction was only observed in the soluble fraction, suggesting that CDK2 phosphorylation occurs when HELB is not bound to chromatin. In addition, because CDK2 was similarly enriched in the WT HELB, D506G HELB, and 3xA HELB soluble samples, this indicates that HELB interaction with CDK2 is independent of interaction with RPA. We also observed cyclin A2 in all five replicates of the WT HELB, D506G HELB, and 3xA HELB soluble samples but zero of the EV samples (Supplementary Table S9), suggesting that HELB also interacts with cyclin A2 in an RPA independent manner. This interaction with cyclin A2 was also detected previously observed in cells treated with neocarzinostatin [23]. However, another study indicated that cyclin E, and not cyclin A, interacts with HELB [25].

Because D506G substitution interfered with HELB interaction with RPA we used affinity purification to confirm the reduction in this important interaction. RPA70 co-purifies with SFB-tagged WT HELB (Fig. 3D and Supplementary Fig. S6), and co-purification of RPA70 with the RPA-interaction deficient SFB-3xA HELB is reduced 20-fold relative to WT HELB (Fig. 3D and Supplementary Fig. S6). Co-purification of RPA70 with SFB-D506G HELB is reduced 5-fold relative to with SFB-WT HELB (Fig. 3D and Supplementary Fig. S6), indicating that substitution of D506 alone is sufficient to interfere with HELB interaction with RPA and that the HELB encoded by the rs75770066 missense allele has impaired interaction with RPA. To quantify this change, we assessed the effect of D506G substitution on the affinity of HELB for RPA by measuring the anisotropy of N-terminally FAM-labeled peptides derived from the HELB HSM incubated with varying concentrations of the RPA70 NTD and found that D506G substitution reduces the affinity for RPA 10-fold (Fig. 3E). Although other regions of HELB may also interact with RPA, the results of these experiments indicate that a D506G substitution interferes with HELB-RPA interaction.

The impaired interaction of D506G HELB with RPA suggests that D506G substitution may interfere with unwinding of RPA-coated ssDNA. This was tested using a substrate with a 70T 5′-ssDNA overhang pre-incubated with RPA. RPA did not have a significant effect on unwinding by WT HELB (Fig. 3F). Unwinding by D506G HELB was also not statistically different than unwinding by WT HELB on naked DNA substrates. Interestingly, unwinding by D506G HELB was reduced ∼3-fold by RPA, suggesting that impaired interaction with RPA impairs the ability of D506G HELB to load onto the RPA-coated 5′-ssDNA tail and initiate unwinding.

### D506G substitution in HELB interferes with HELB localization to chromatin

**Figure 4.**
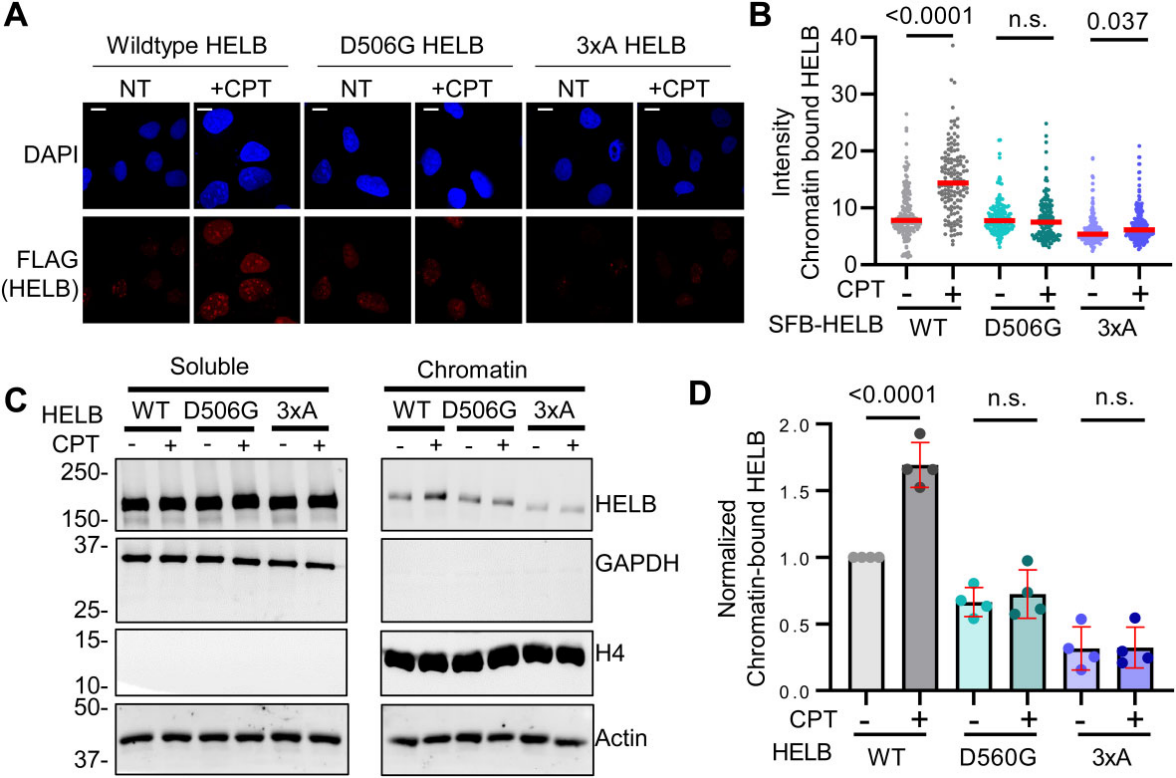
D506G substitution in HELB interferes with localization of HELB to chromatin in response to replication stress. (**A**) HELB^KO^ U2OS cells expressing SFB-HELB variants (WT, D506G, and 3xA) were treated with 10 μM CPT for 1 h followed by pre-extraction to remove nonchromatin-bound proteins before fixing and staining with an anti-FLAG antibody to detect SFB-HELB. (**B**) The intensity of HELB in the nuclei of >130 cells for each condition was quantified using CellProfiler [47] from biological triplicate experiments. Scale bar indicates 1 μm. Significance was assessed using a one-way ANOVA; n.s. is not significant. (**C**) Lysates of HELB^KO^ HEK293T cells expressing SFB-HELB variants (WT, D506G, and 3xA) with or without CPT treatment were separated into soluble and chromatin fractions and visualized by western blotting. Additional replicates are shown in Supplementary Fig. S7. (**D**) Quantification of western blots in panel (C) and in Supplementary Fig. S7.

HELB localizes to foci on chromatin during DNA replication stress and DNA damage [21, 22, 25]. Localization of HELB to sites of laser induced microirradiation is dependent on its interaction with RPA as 3xA HELB is severely impaired in recruitment to sites of microirradiation [23]. The necessity of interaction with RPA for recruitment of HELB to other types of DNA damage is unknown. We measured HELB localization to chromatin by immunofluorescence in HELB^KO^ U2OS cells expressing SFB-tagged HELB variants treated with CPT, a topoisomerase 1 inhibitor that induces DNA replication stress (Fig. 4A and B). WT SFB-HELB bound to chromatin increases with CPT treatment while SFB-D506G HELB bound to chromatin does not increase. A very small increase in SFB-3xA HELB is observed with CPT treatment, but the level of chromatin-bound HELB is still below that of untreated cells expressing WT HELB. We also found that WT-HELB localized to chromatin with CPT treatment by chromatin fractionation, but neither D506G nor 3xA HELB was recruited to chromatin with CPT treatment (Fig. 4C and D, and Supplementary Fig. S7). This indicates that interaction with RPA is required for localization of HELB to DNA with replication stress and that the missense variant encoded by rs75770066 does not localize to DNA with DNA replication stress.

**Figure 5.**
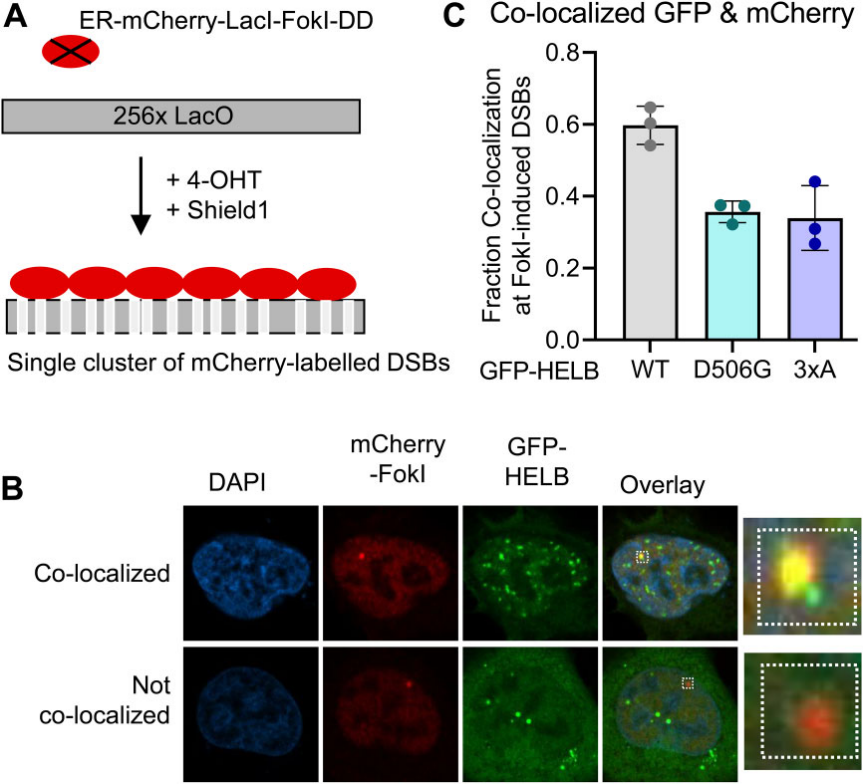
D506G HELB has reduced co-localization with DSBs relative to WT HELB. (**A**) A single array of mCherry labeled DSBs is produced upon treatment of U2OS DSB reporter cells with 4-OHT and Shield1. (**B**) DSB reporter cells expressing GFP-HELB variants (WT, D506G, and 3xA) were treated with 1 μM 4-OHT and 1 μM Shield1 for 4 h. Cells were fixed and stained with an antibody to mCherry before imaging. (**C**) Localization of GFP-HELB relative to Cherry-FokI foci was quantified blind for >150 cells per condition from biological triplicate experiments. Significance was assessed using a two-way ANOVA; n.s. is not significant.

We also measured the ability of HELB variants to localize to DSBs using a DSB reporter [39]. The DSB reporter cells stably express an mCherry-LacI-FokI fusion protein that creates DSBs at a single locus on chromosome 1 containing 256 copies of the lac operator [48]. The fusion protein also expresses a modified estradiol receptor for nuclear targeting and a destabilization domain to prevent unwanted DSBs [39]. Following treatment of DSB reporter cells with 4-OHT and Shield1, the fusion protein is stabilized and DSBs are induced (Fig. 5A). GFP-tagged HELB variants were expressed in the DSB reporter cells and co-localization of mCherry-FokI and GFP-HELB was measured. Approximately 60% of the mCherry-FokI foci co-localize with WT GFP-HELB (Fig. 5B and C, and Supplementary Video SV1). The lack of co-localization of GFP-HELB with mCherry-FokI in all cells could be due to the cell cycle dependent localization of HELB [23, 25]. Co-localization of both GFP-D506G HELB and GFP-3xA HELB is reduced to ∼30% (Fig. 5B and C). A corresponding increase in the fraction of mCherry-FokI foci that do not co-localize with HELB is observed in cells expressing GFP-D506G HELB and GFP-3xA HELB (Fig. 5B and C, Supplementary Video V2). This indicates that interaction with RPA promotes HELB localization to DSBs. In addition, recruitment of the D506G variant is reduced relative to WT HELB.

### D506G substitution in HELB increases repair of DSBs by HR

**Figure 6.**
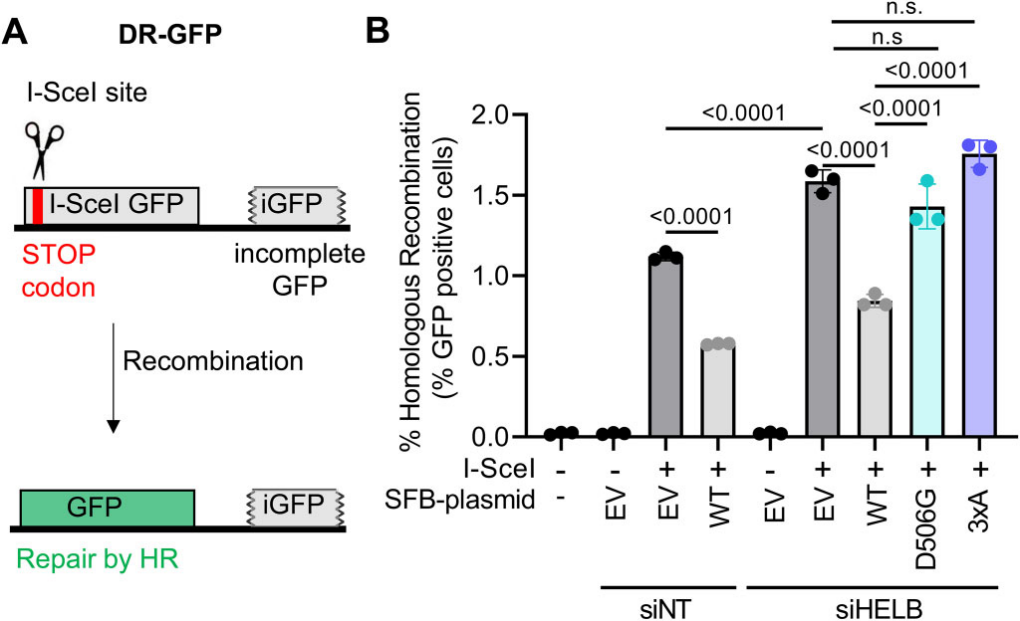
Expression of D506G HELB increases HR levels relative to expression of WT HELB. (**A**) Expression of I-SceI in DR-GFP-U2OS cells produces a DSB in the I-SceI GFP gene. Cells that repair the DSB by HR are identified as GFP-positive cells by the flow cytometry. (**B**) The percentage of GFP-positive cells in each sample are plotted. Significance was assessed using a one-way ANOVA; n.s. is not significant.

The impaired localization of D506G HELB to DSBs suggests regulation of HR by HELB [22, 23] may also be impaired. We measured recombination in U2OS cells containing a DR-GFP reporter [49]. This reporter contains a direct repeat of two *GFP* genes: *I-SceI GFP* contains the recognition site for I-SceI endonuclease and two stop codons and *iGFP* is an internal fragment of the *GFP* gene that can be used as a repair template during HR, restoring the *GFP* gene (Fig. 6A). GFP positive cells were absent in cells not transfected with a plasmid encoding I-SceI (Fig. 6B). Transfection with plasmids encoding I-SceI and SFB-EV results in ∼1.2% GFP positive cells. Consistent with previous results [23], we observed a 50% reduction in GFP positive cells when transfecting with plasmids encoding I-SceI and WT HELB (Fig. 6B). Similarly, we saw an increase in GFP positive cells when we transfected with siRNA targeting HELB and with plasmids encoding I-SceI and EV. No increase in GFP positive cells was observed when cells were transfected with siRNA targeting HELB and a plasmid encoding WT HELB that rescued knockdown of HELB expression. However, neither expression of D506G HELB nor 3xA HELB could rescue knockdown of HELB expression; in both cases, recombination rates were increased similarly to those of cells with knockdown of HELB expression and expression of the EV. Therefore, interaction with RPA is required for HELB to limit HR, and the D506G missense variant caused by rs75770066 interferes with HELB’s ability to inhibit HR.

## Discussion

There are multiple SNPs in *HELB* that are associated with altered ANM in women. In addition, multiple SNPs in *HELB* are associated with reproductive traits in male and female cattle [50, 51], suggesting HELB plays a critical role in reproductive fitness. In humans, rs75770066 is a low frequency variant resulting in a D506G substitution in the protein that alters ANM [8, 14–16]. We compared the enzymatic activity, interaction partners and cellular activity of D506G HELB to WT HELB to gain insight into how rs75770066 may alter ANM. Although D506 is located in the HSM insertion within the helicase domain of HELB (Fig. 1A–C), D506G substitution does not impair the helicase activity of HELB on naked DNA (Fig. 2). The HSM partially overlaps with an acidic motif previously described in HELB [21] and contains three negatively charged amino acids (E499, D506, and D510) previously shown to interact with RPA [21, 23, 28]. Consistent with this prior data, AlphaFold Multimer [26, 29] predicts that the HSM interacts with the basic cleft on the RPA70 NTD (Fig. 1D). The HSM is an insertion within the 1A domain of HELB. In SF1B helicases like HELB that unwind DNA with a 5′-to-3′ polarity, the 1A domain is the leading edge of the enzyme [52–57]. This positions the RPA interacting region in front of HELB, which is consistent with the ability of HELB to clear RPA from ssDNA [24].

We found that WT HELB interacts with RPA in both the chromatin and soluble fractions (Fig. 3 and Supplementary Figs S4 and S5, and Supplementary Tables S4–S7), suggesting that HELB does not require DNA to interact with RPA. This possibility is consistent with structural data indicating interaction of a peptide of HELB including E499, D506, and D510 with the RPA70 NTD in the absence of DNA [21, 28]. D506G substitution reduces interaction of HELB with several proteins including all three subunits of RPA (Fig. 3). *In vitro* D5606G substitution in a peptide comprised of amino acids 493–519 of HELB reduces the affinity for the RPA70 NTD by ∼10-fold, indicating that this single amino acid change greatly interferes with HELB interaction with RPA. Additionally, although unwinding of naked DNA is not affected by D506G substitution, D506G reduces the rate of HELB unwinding of RPA-coated DNA (Fig. 3F). This suggests that D506G HELB unwinding activity would be limited in the cell by the binding of RPA to ssDNA.

Unlike interaction with RPA, D506G substitution does not affect interaction with CDK2 or cyclin A2 (Supplementary Fig. S5), suggesting that the phosphorylation of HELB at the G1 to S transition [25] is mediated by CDK2 in complex with cyclin A2. This interaction with cyclin A2 was also observed previously in cells treated with the radiomimetic drug neocarzinostatin [23]. However, another study found that cyclin E co-immunoprecipitated with HELB whereas cyclin A did not [25]. Cyclin E2 was present in both our chromatin and soluble input samples but neither of the TAP samples, whereas cyclin A2 was similarly present in both the chromatin and soluble input samples but notably also enriched in the soluble TAP (Supplementary Table S9), suggesting HELB interacts with cyclin A2.

Interestingly, several HELB interacting proteins identified by TAP-MS do not appear to interact directly with HELB but, instead, seem to be in a complex containing HELB and RPA (Fig. 3, and Supplementary Figs S4 and S5, and Supplementary Tables S8 and S9). SMARCAL1, YBX1, HMCES, and BLM are all significantly enriched in all five replicates of TAP-MS with WT HELB but are not significantly enriched in any of the five replicates of TAP-MS with 3xA HELB (Supplementary Tables S8 and S9). These proteins are all involved in DNA repair and the DNA replication stress response [58–61], and additional proteins involved in DNA repair are enriched to a greater degree in the WT HELB sample than the 3xA HELB sample. This suggests that HELB functions in these processes as a component of a multiprotein complex with the interactions mediated through RPA.

In cells, HELB performs several important functions that may affect cell growth and viability. HELB promotes timely entry into S phase [20, 25], enhances recovery from DNA replication stress [21], and regulates HR [22, 23]. Recruitment of HELB to sites of laser induced microirradiation has previously been shown to be dependent on interaction with RPA [23]. In cells expressing RPA-interaction-deficient 3xA HELB, we found that recruitment of HELB to chromatin in response to replication stress (Fig. 4) and recruitment to DSBs (Fig. 5) are also dependent on interaction with RPA. In addition, we found that interaction with RPA is required for HELB to inhibit HR (Fig. 6). In their previous report that HELB inhibits HR, the Durocher group showed that HELB reduces long-range end resection by EXO1 and BLM-DNA2 [23]. They found that inhibition of end-resection required interaction with RPA as cells expressing the 3xA HELB variant had similar levels of ssDNA produced by resection as cells lacking HELB [23], suggesting that interaction with RPA is required for HELB to inhibit HR. The results in Fig. 6 confirm that this is indeed the case.

D506G substitution also interferes with the recruitment of HELB to chromatin with DNA replication stress (Fig. 4) and to sites of DSBs (Fig. 5) likely because D506G substitution reduces HELB interaction with RPA. For localization to DSBs (Fig. 5), recruitment of D506G HELB is similar to recruitment of 3xA HELB even though interaction of D506G HELB with RPA is not impaired to the same extent as interaction of 3xA with RPA (Fig. 3 and Supplementary Fig. S6). This indicates that a strong interaction with RPA is required for HELB localization to DSBs. Because we found that D506G interferes with HELB recruitment to DSBs, it is not surprising that D506G HELB is unable to suppress HR at DSBs. Similar to recruitment, even though interaction with RPA is not reduced to the same level in cells expressing D506G HELB as 3xA HELB, HR levels in the cells expressing D506G HELB are the same as in cells with expression of HELB knocked down with siRNA or in cells expressing 3xA HELB (Fig. 6), indicating D506G HELB is unable to suppress HR.

HELB has been reported to both stimulate [22] and inhibit [23] HR at DSBs. One group [22] measured HR in SW480/SN.3 colon carcinoma cells containing a SCneo recombination reporter [62]. The other group [23] measured HR in HeLa cervical carcinoma cells containing a DR-GFP recombination reporter [49]. Although the two groups used different reporter assays, both are based on repair of an I-SceI induced DSB by HR, suggesting the different outcomes are not likely due to the different reporter assays. The group that observed inhibition of HR by HELB [23] transfected with siRNA targeting HELB 24 h prior to transfection with a plasmid encoding I-SceI while the group that observed HELB enhanced HR [22] transfected with shRNA targeting HELB and a plasmid encoding I-SceI simultaneously. Although both groups observed knockdown of HELB expression 48 h after transfection with plasmid, it is unlikely that knockdown of HELB expression occurred on the same timescale as I-SceI production in the experiments that observed HELB enhanced HR [22]. However, this is also not the likely cause of the contrasting results because insufficient knockdown of HELB expression would likely have no effect on recombination levels instead of producing the opposite effect. It is possible that the different cell lines are the cause of the disparate results. We measured HR levels in a third human cell line, U2OS osteosarcoma cells containing the DR-GFP reporter and observed inhibition of HR by HELB and a corresponding increase in HR in cells depleted of HELB, as was observed by [23]. Thus, in at least two human cell lines, HELB is a negative regulator of HR.

**Figure 7.**
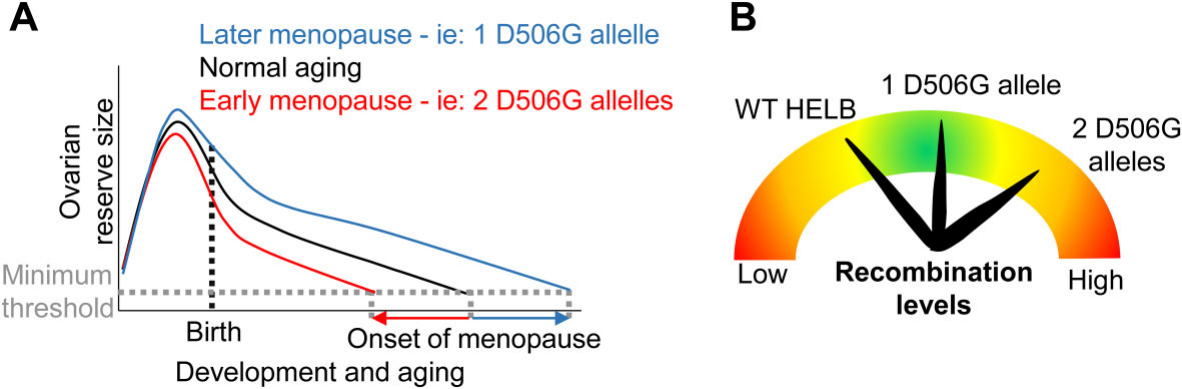
Model for D506G HELB effect on age at menopause. (**A**) Menopause occurs when oocyte levels drop below a minimum threshold. A single D506G allele may increase the size of the ovarian reserve resulting in delayed ANM while two D506G alleles may decrease the size of the ovarian reserve resulting in early ANM. (**B**) Repair of DSBs by recombination is essential for pairing and segregation of homologous chromosomes during meiosis. One allele encoding D506G HELB may enhance recombination and increase oocyte viability, but a second D506G allele may increase recombination enough that genomic rearrangements occur, leading to diminished ovarian reserves and early ANM.

Because natural menopause occurs when the oocyte pool is depleted below the threshold necessary for proper ovarian function, changes in the size of the oocyte pool due to decreased survival with replication stress, altered recombination levels, or changes in DNA repair may lead to changes in ANM due to impaired establishment or maintenance of the ovarian reserve. The size of the ovarian reserve is fixed soon after birth by clearing of defective oocytes during the perinatal period [63, 64] (Fig. 7A). Among the reported cellular functions of HELB, we can envision how only an increase in recombination levels caused by D506G HELB has the potential to affect oocyte viability negatively or positively (Fig. 7B). Repair of programed meiotic DSBs by recombination is essential for chromosome pairing via crossover formation and proper segregation in meiosis I. Because D506G HELB is unable to inhibit HR, one allele encoding D506G HELB may enhance recombination and increase oocyte viability. However, a second D506G allele may increase recombination to the point that genomic rearrangements occur, leading to diminished ovarian reserves and early ANM. Interestingly, both insufficient crossover formation and excess crossover formation lead to impaired chromosome segregation, which results in aneuploidy [65, 66]. In addition, excessive recombination causes nonallelic HR, resulting in genomic rearrangements [67]. Because both aneuploidy and nonallelic HR reduce the viability of the oocyte, either too much recombination or too little recombination will reduce the size of the oocyte pool. Failure to correctly repair meiotic DSBs by recombination activates the checkpoint kinases, CHK1 and CHK2, leading to apoptosis [63, 68]. In humans, <10% of oocytes that begin meiosis survive to puberty [69]. This suggests that failure to correctly repair meiotic DSBs by recombination would reduce the size of the ovarian reserve, leading to premature menopause. In addition, ageing oocytes have reduced BRCA1 levels and rely more on DNA repair by error-prone pathways including NHEJ [70]. The BRCA1-independent HR observed in Brca1^−/−^ p53^−/−^ mouse mammary tumor cells depleted of Helb [23] suggests that aged oocytes expressing D506G HELB may have higher levels of error-free repair by HR than WT oocytes. Thus, the inability of D506G HELB to regulate HR may result in altered ANM caused by rs75770066.

Interestingly, later menopause correlates with increased lifespan [71, 72] while early menopause is associated with early mortality [73, 74]. These differences are not due to changes in estrogen levels as hormone replacement does not alter menopause timing [74], suggesting that changes in ANM are due to alterations in the rate of aging [71]. This suggests that women (and likely also men) who are heterozygous for rs75770066 will on average have a longer lifespan than those with the common allele.

## Supplementary Material

ugaf019_Supplemental_Files

## Data Availability

The AlphaFold model is available at ModelArchive (https://www.modelarchive.org/doi/10.5452/ma-7ry8k/). Proteomics data has been deposited in the MassIVE repository (dataset: MSV000096390) and is available at ProteomeXchange (dataset: PXD057796).
