# Supplementary material for: Rare SNP in the HELB gene interferes with RPA interaction and cellular function of HELB
Source: NAR Mol Med. 2025 May 23;2(2):ugaf019. doi: 10.1093/narmme/ugaf019 (PMC12147029; doi:10.1093/narmme/ugaf019)
Supplement: ugaf019_Supplemental_Files [file ugaf019_Supplemental_Files.zip › D506G Supplement 250514.pdf]

Supplementary information for:

Rare SNP in the *HELB* gene interferes with RPA interaction and cellular function of HELB

By Bertha Osei, Benjamin H. May, Joseph S. Beard, Matthew D. Thompson, Duah Alkam, Maroof Khan Zafar, Erik Bergstrom, Stephanie D. Byrum, Eric J. Enemark, Kirk L. West, and Alicia K. Byrd

Supplementary Table S1. Oligonucleotide sequences

Name	Sequence (5′-to-3′)*
30T-30bp fork	/56-FAM/T_{30}CTGACTGCGATCCGACTGTCCTGCATGATG<br>CATCATGCAGGACAGTCGGATCGCAGTCAGT_{10}
70T-30bp fork	/56-FAM/T_{70}CTGACTGCGATCCGACTGTCCTGCATGATG<br>CATCATGCAGGACAGTCGGATCGCAGTCAGT_{10}
Annealing trap	CTGACTGCGATCCGACTGTCCTGCATGATG

*Duplex forming regions are underlined.

Supplementary Table S2. sgRNA sequences

Name	Sequence (5′-to-3′)*
HELB gRNA 1	GGATTTGCTGCTGCACCTCCTCC
HELB gRNA 2	GGAGCCGCAGCTACTTGTGCCTG

*PAM sequences are underlined.

Supplementary Table S3. Peptide sequences

Name	Sequence (5′-to-3′)*
WT HELB_493-919	(6-FAM)EQLEEREVKKACEDFEQDQNASEEWIT
D506G HELB_493-919	(6-FAM)EQLEEREVKKACEGFEQDQNASEEWIT

Supplementary Tables S4-S7 are available as an Excel workbook.

Supplementary Table S8. Proteins identified in SFB-HELB chromatin fraction samples that were not identified in SFB-EV.

		Average Log2 Intensity			
Gene	Description	EV	WT	D506G	3xA
PRKAR2A	Protein kinase cAMP-dependent regulatory type II alpha	0	8.83	5.94	2.84
RCC2	Epididymis secretory sperm binding protein	0	7.67	3.00	2.72
HMBS	hydroxymethylbilane synthase	0	8.73	5.47	0
CUL4B	Cullin 4B	0	7.11	2.49	2.54
MAP4	Microtubule-associated protein	0	5.75	7.96	2.46
SMARCAL1	SWI/SNF-related matrix-associated actin-dependent regulator of chromatin subfamily A-like protein 1	0	**15.1**	**12.5**	0
IMPDH	Inosine-5'-monophosphate dehydrogenase	0	4.75	8.46	2.79
CEP170	Centrosomal protein 170kDa	0	11.5	10.3	3.60
RADX	RPA-related protein RADX	0	9.97	2.05	0
MYO1B	MYO1B variant protein	0	9.71	0	0
cDNA FLJ14222 fis	highly similar to Nucleolar complex protein 2 homolog	0	**19.3**	7.64	7.44
AP1G1	AP-1 complex subunit gamma	0	9.51	0	0
OLA1	Obg-like ATPase 1	0	4.33	6.43	4.50
PIK3C2A	Phosphatidylinositol-4-phosphate 3-kinase, catalytic subunit type 2 alpha	0	2.54	9.68	5.74
RPLP1	Large ribosomal subunit protein P1	0	5.30	11.2	3.30
HSP90AB1	Heat shock protein HSP 90-beta	0	10.0	9.27	5.36
PGAM1	Phosphoglycerate mutase 1	0	7.97	2.12	2.44
PRDX6	Peroxiredoxin-6	0	9.35	5.29	2.40
PRDX2	Peroxiredoxin-2	0	1.95	8.85	6.37
YBX1	Y-box-binding protein 1	0	**13.8**	**12.1**	0
RPLP1	RPLP1 protein	0	2.67	7.02	0
RHOU	Rho-related GTP-binding protein RhoU	0	**17.8**	13.7	**16.0**
HUWE1	E3 ubiquitin-protein ligase HUWE1	0	**19.6**	12.8	8.14
HMCES	Abasic site processing protein HMCES	0	**13.7**	5.02	0

Intensities in **bold** indicate conditions in which the protein was detected in all 5 replicates.
Intensities in gray indicate conditions in which the protein was detected in <3 of the 5 replicates.

Supplementary Table S9. Proteins identified in SFB-HELB soluble fraction samples that were not identified in SFB-EV.

		Average Log2 Intensity			
Gene	Description	EV	WT	D506G	3xA
BAG5	BCL2-associated athanogene 5	0	**13.4**	**12.7**	5.16
SKP2	S-phase kinase associated protein 2	0	**17.2**	**16.9**	**16.2**
ELOB	Epididymis secretory sperm binding protein	0	**13.3**	**13.6**	10.8
cDNA FLJ75589	highly similar to Homo sapiens mutS homolog 3 (E. coli) (MSH3)	0	7.66	0	0
BLM	RecQ-like DNA helicase BLM	0	**13.1**	0	0
DIP2A	Disco interacting protein 2 homolog A	0	11.4	0	0
RPL19	Ribosomal protein L19	0	8.12	8.29	8.06
PNKD	PNKD metallo-beta-lactamase domain containing	0	5.65	8.44	0
RAF1	non-specific serine/threonine protein kinase	0	9.96	13.4	3.11
SMARCAL1	SWI/SNF-related matrix-associated actin-dependent regulator of chromatin subfamily A-like protein 1	0	**16.3**	**13.0**	0
RPL24	Ribosomal protein L24	0	**13.5**	**13.5**	11.1
CEP170	Centrosomal protein 170kDa	0	17.3	17.5	**20.7**
MVB12A	Multivesicular body subunit 12A	0	9.58	9.71	6.00
RPL13	60S ribosomal protein L13	0	11.0	13.5	12.7
SORT1	cDNA FLJ76489, highly similar to Homo sapiens sortilin 1	0	**15.6**	**15.2**	**14.3**
cDNA FLJ14222 fis	highly similar to Nucleolar complex protein 2 homolog	0	12.4	8.15	4.44
AIP	peptidylprolyl isomerase	0	5.72	8.52	0
LIMD1	LIM domain containing 1	0	8.73	5.79	5.92
RPL32	Ribosomal protein L32	0	5.17	7.12	2.51
ELOC	Elongin-C	0	8.26	10.6	0
RPL30	Large ribosomal subunit protein eL30	0	12.0	14.8	13.8
FAU	FAU ubiquitin like and ribosomal protein S30 fusion	0	11.0	10.6	7.97
RPL26	Large ribosomal subunit protein RPL26	0	**13.5**	**13.2**	8.07
S100A8	Protein S100-A8	0	3.00	8.63	0
RPL35A	Large ribosomal subunit protein eL33	0	8.16	11.2	5.66
CCNA2	Cyclin-A2	0	**17.7**	**17.8**	**16.5**
DNAJB1	DnaJ homolog subfamily B member 1	0	7.01	2.63	2.67
HMGB2	High mobility group protein B2	0	6.26	3.02	8.13
RPL10	Large ribosomal subunit protein L10	0	10.1	12.6	12.5
RFC1	Replication factor C subunit 1	0	10.1	2.12	0
MKI67	Proliferation marker protein Ki-67	0	10.5	10.8	13.9
RPS9	Small ribosomal subunit protein uS4	0	**14.1**	**14.1**	**13.3**
RPL34	Large ribosomal subunit protein eL34	0	11.7	14.5	8.12
METAP1	Methionine aminopeptidase 1	0	8.85	6.76	2.11
CKS1B	Cyclin-dependent kinases regulatory subunit 1	0	**15.0**	**14.9**	**13.8**
WDR5	WD repeat-containing protein 5	0	**13.1**	**12.9**	5.37
XPC	Xeroderma pigmentosum, complementation group C	0	7.27	0	0
TOP3A	DNA topoisomerase 3-alpha	0	10.1	0	0
COX17	Cytochrome c oxidase copper chaperone	0	16.6	16.5	**19.7**
WRN	Bifunctional 3'-5' exonuclease/ATP-dependent helicase WRN	0	8.22	0	0
TUBB8	Tubulin beta-8 chain	0	7.55	12.6	4.46
HSDL2	Hydroxysteroid dehydrogenase-like protein 2	0	11.4	0	0
SCAI	Protein SCAI	0	**14.2**	**14.6**	**13.6**
DYNC2LI1	Cytoplasmic dynein 2 light intermediate chain 1	0	9.35	9.49	3.32
TRIM41	E3 ubiquitin-protein ligase TRIM41	0	9.23	12.0	6.24
HMCES	Abasic site processing protein HMCES	0	**15.0**	**13.0**	0
ZNF512B	Zinc finger protein 512B	0	9.73	10.0	13.1
TMX4	Thioredoxin-related transmembrane protein 4	0	7.57	15.1	7.59
PRTFDC1	Phosphoribosyltransferase domain-containing protein 1	0	0	8.09	2.65
RPL36	Large ribosomal subunit protein eL36	0	10.4	**13.5**	8.07

Intensities in **bold** indicate conditions in which the protein was detected in all 5 replicates.
Intensities in gray indicate conditions in which the protein was detected in <3 of the 5 replicates.

Supplementary Figure S1

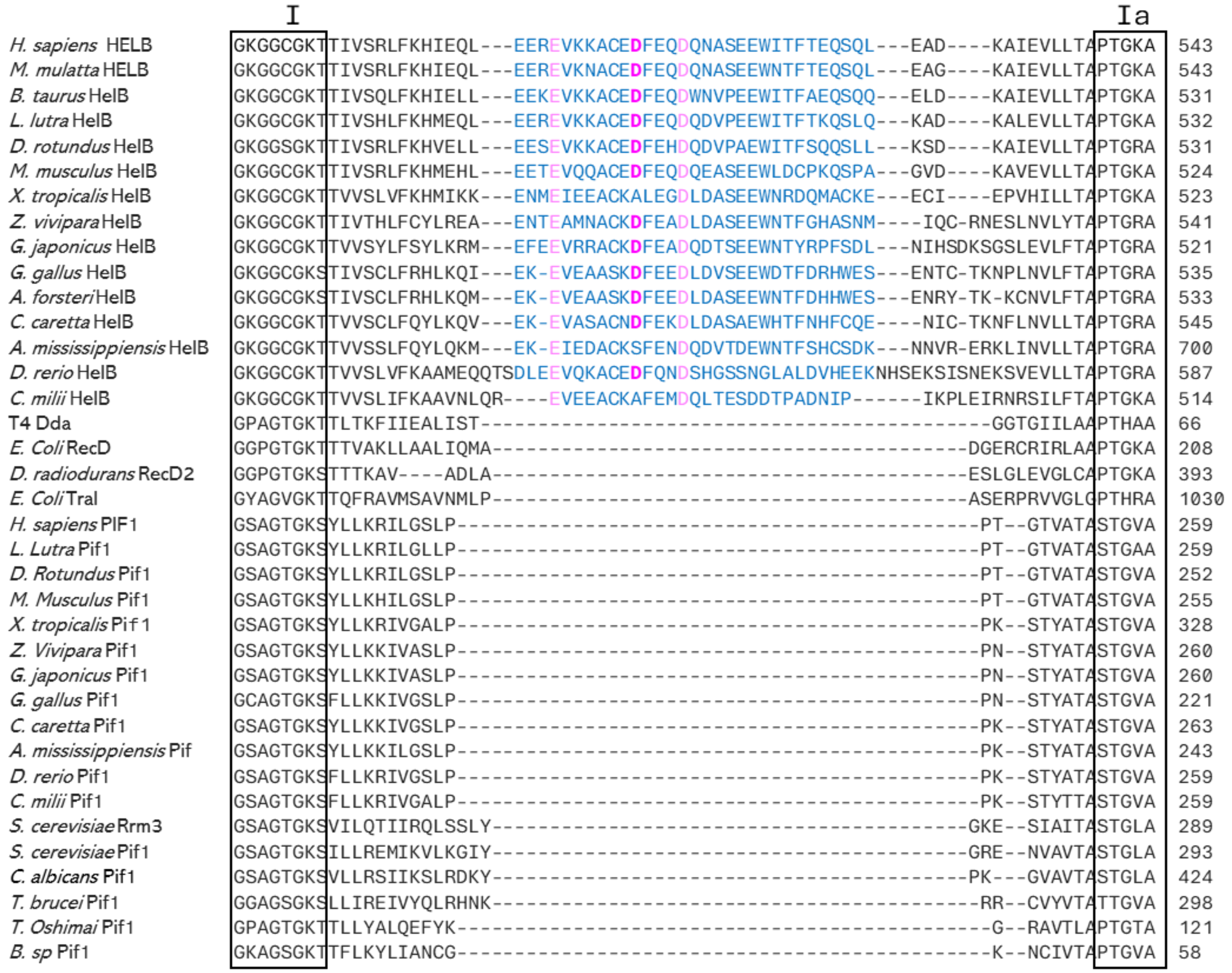

Supplemental Figure S1. HELB family proteins contain a HELB specific motif (HSM). Sequence alignments of *Homo sapiens* HELB, *Macaca mulatta* HELB, *Bos taurus* HelB, *Lutra lutra* HelB, *Desmondus rotundus* HelB, *Mus musculus* HelB, *Xenopus tropicalis* HelB, *Zootoca vivipara* HelB, *Gekko japonicus* HelB, *Gallus gallus* HelB, *Aptenodytes forsteri* HelB, *Caretta caretta* HelB, *Alligator mississippiensis* HelB, *Danio rerio* HelB, *Callorhinchus milii* HelB, Bacteriophage T4 Dda, *E. coli* RecD, *Deinococcus radiodurans* RecD2, *E. coli* TraI, *Homo sapiens* PIF1, *Lutra lutra* Pif1, *Desmondus rotundus* Pif1, *Mus musculus* Pif1, *Xenopus tropicalis* Pif1, *Zootoca vivipara* Pif1, *Gekko japonicus* Pif1, *Gallus gallus* Pif1, *Caretta caretta* Pif1, *Alligator mississippiensis* Pif1, *Danio rerio* Pif1, *Callorhinchus milii* Pif1, *Saccharomyces cerevisiae* Rrm3, *Saccharomyces cerevisiae* Pif1, *Candida albicans* Pif1, *Trypanosoma brucei* Pif1, *Thermus oshimai* Pif1, and *Bacteroides sp* Pif1 helicases by Clustal Omega shows a HELB specific motif (HSM) (blue) exists between helicase motifs I and Ia. E499, D506, and D510 (magenta) are located within this HSM.

Supplementary Figure S2

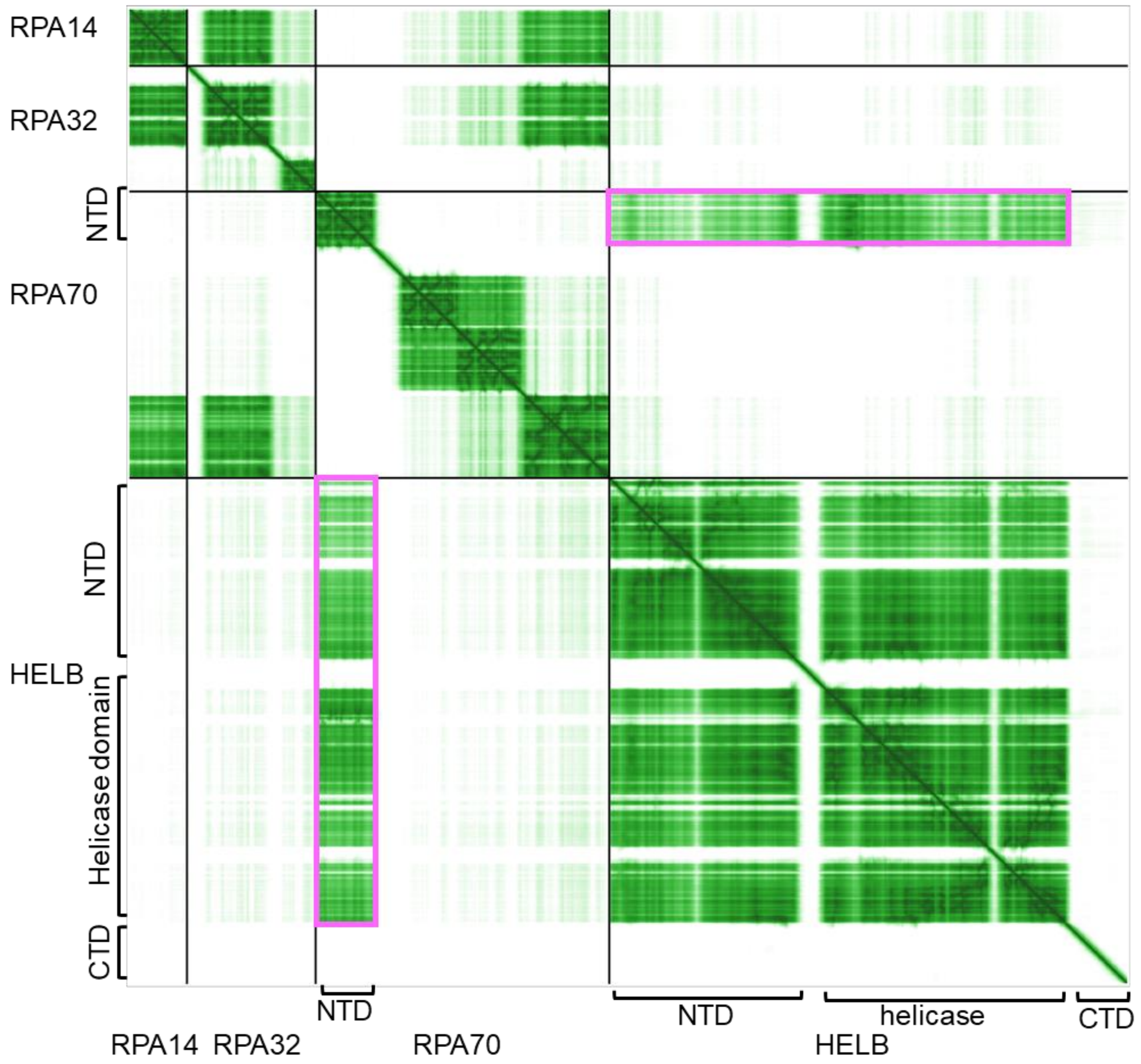

Supplemental Figure S2. The AlphaFold Multimer Predicted Aligned Error (PAE) (1, 2) for RPA14, RPA32, RPA70, and HELB shows interactions between the RPA70 NTD (pink) and HELB are the sole high-confidence interactions between HELB and RPA. Lower PAE is indicated in darker green as plotted by ChimeraX (1–3).

Supplementary Figure S3

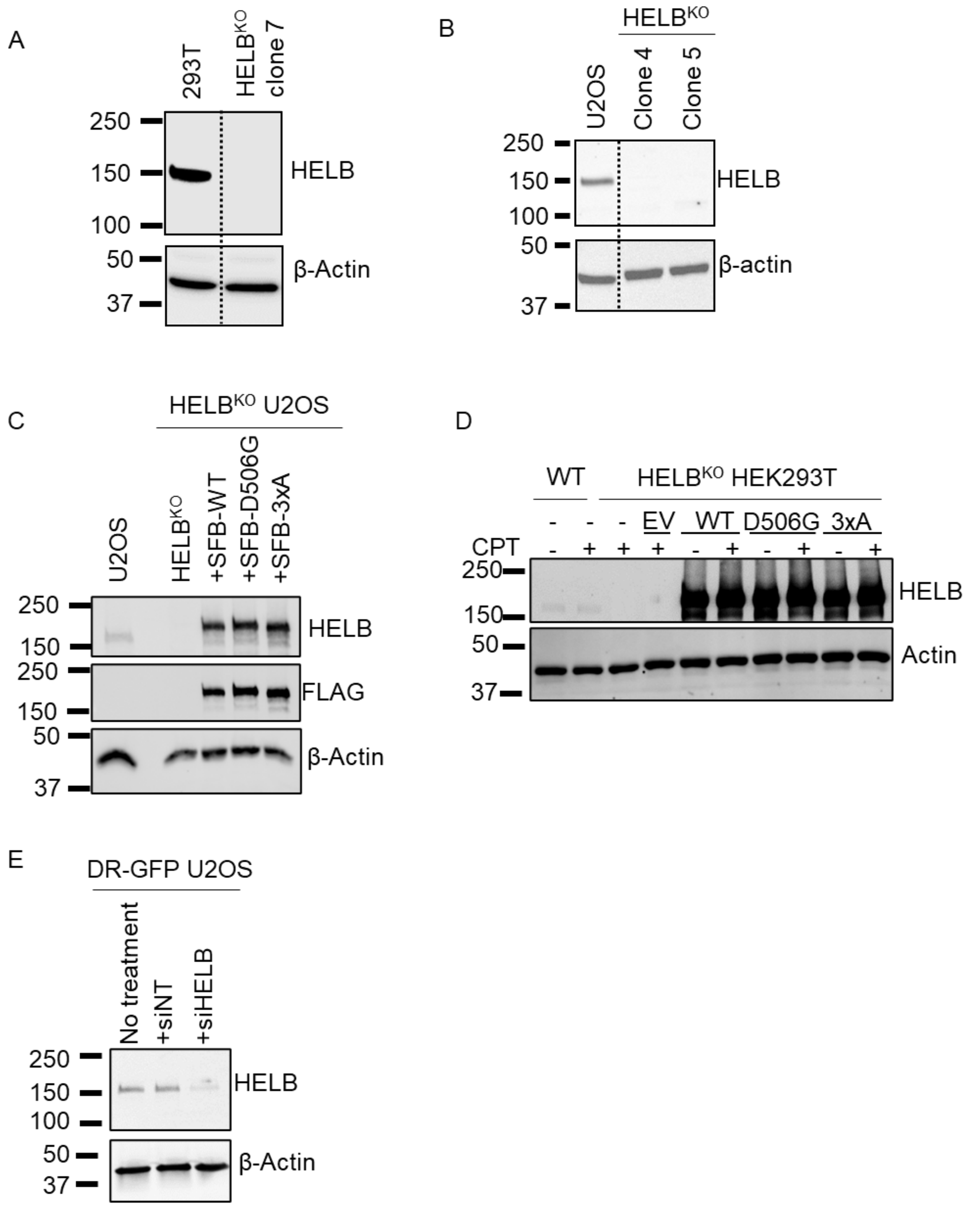

Supplementary Figure S3. Western blots illustrating knockout of HELB and knockdown of HELB expression (**A**) Western blot of 293T cells and HELB^{KO} 293T cells. (**B**) Western blot of U2OS cells and HELB^{KO} U2OS cells. (**C**) Western blot showing expression of SFB-HELB variants in HELB^{KO} U2OS cells. (**D**) Western blot showing expression of SFB-HELB variants in the whole cell extracts of HELB^{KO} HEK293T cells. (**E**) Western blot of DR-GFP-U2OS cells treated with non-targeting siRNA (siNT) or siRNA against HELB (siHELB).

Supplementary Figure S4

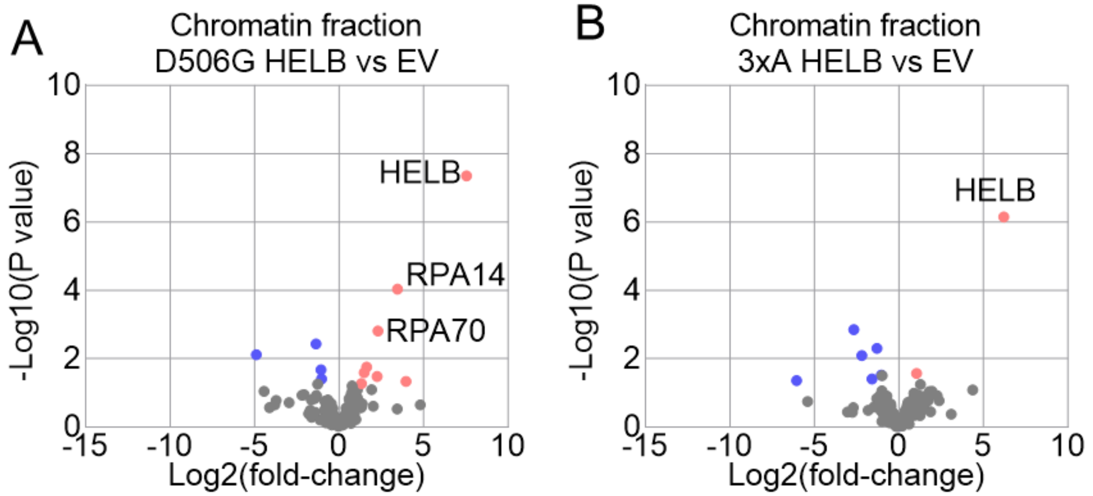

Supplementary Figure S4. D506G HELB and 3xA HELB interacting proteins in the chromatin fraction. Proteins were identified by TAP-MS from the chromatin fraction isolated from $HELB^{KO}$ 293T cells expressing SFB-EV or SFB-HELB (D506G, or 3xA). Significantly enriched (red) and depleted (blue) proteins are plotted for D506G HELB relative to EV (**A**) and 3xA HELB relative to EV (**B**).

Supplementary Figure S5

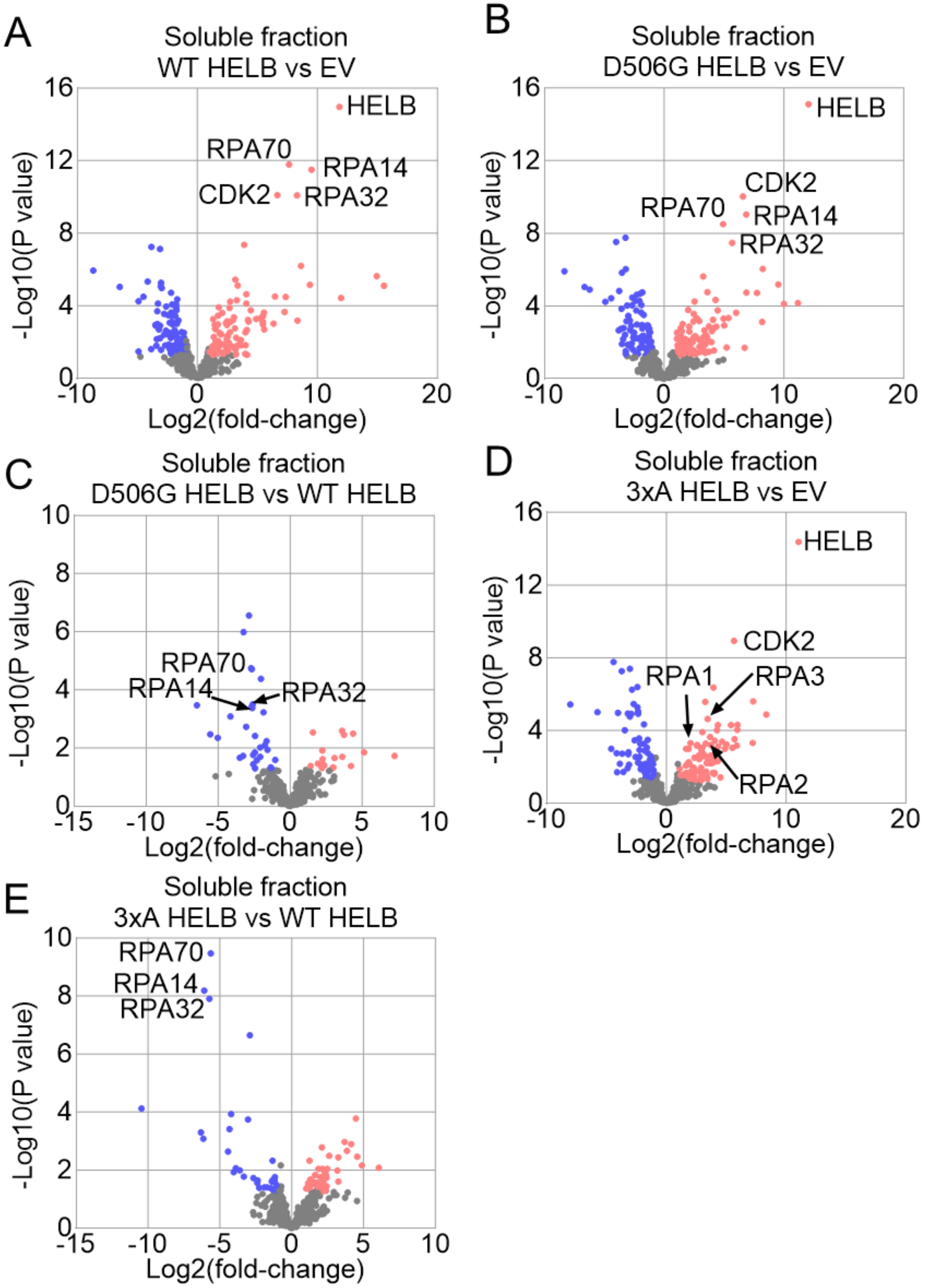

Supplementary Figure S5. D506G HELB and 3xA HELB have reduced interactions with RPA in the soluble fraction relative to WT HELB. Proteins were identified by TAP-MS from soluble fractions isolated from HELB^{KO} 293T cells expressing SFB-EV or SFB-HELB (WT, D506G, or 3xA). Significantly enriched (red) and depleted (blue) proteins are plotted for WT HELB relative to EV (**A**), D506G HELB relative to EV (**B**), D506G HELB relative to WT HELB (**C**), 3xA HELB relative to EV (**D**), and 3xA HELB relative to WT HELB (**E**).

Supplementary Figure S6

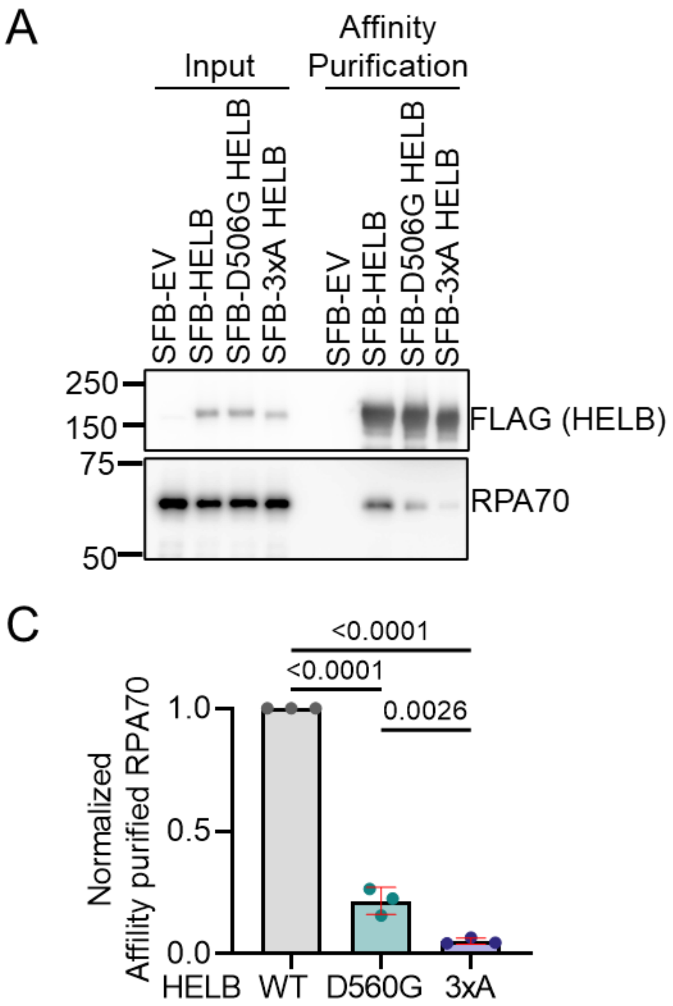

Supplementary Figure S6. D506G substitution in HELB reduces interaction with RPA. (**A-B**) Additional replicates of samples from **Figure 3D** (input and after affinity purification of SFB-HELB variants) on streptavidin beads were probed for FLAG and RPA70 by western blot. (**C**) Quantification of western blots in (**A-B)** and in **Figure 3D**.

Supplementary Figure S7

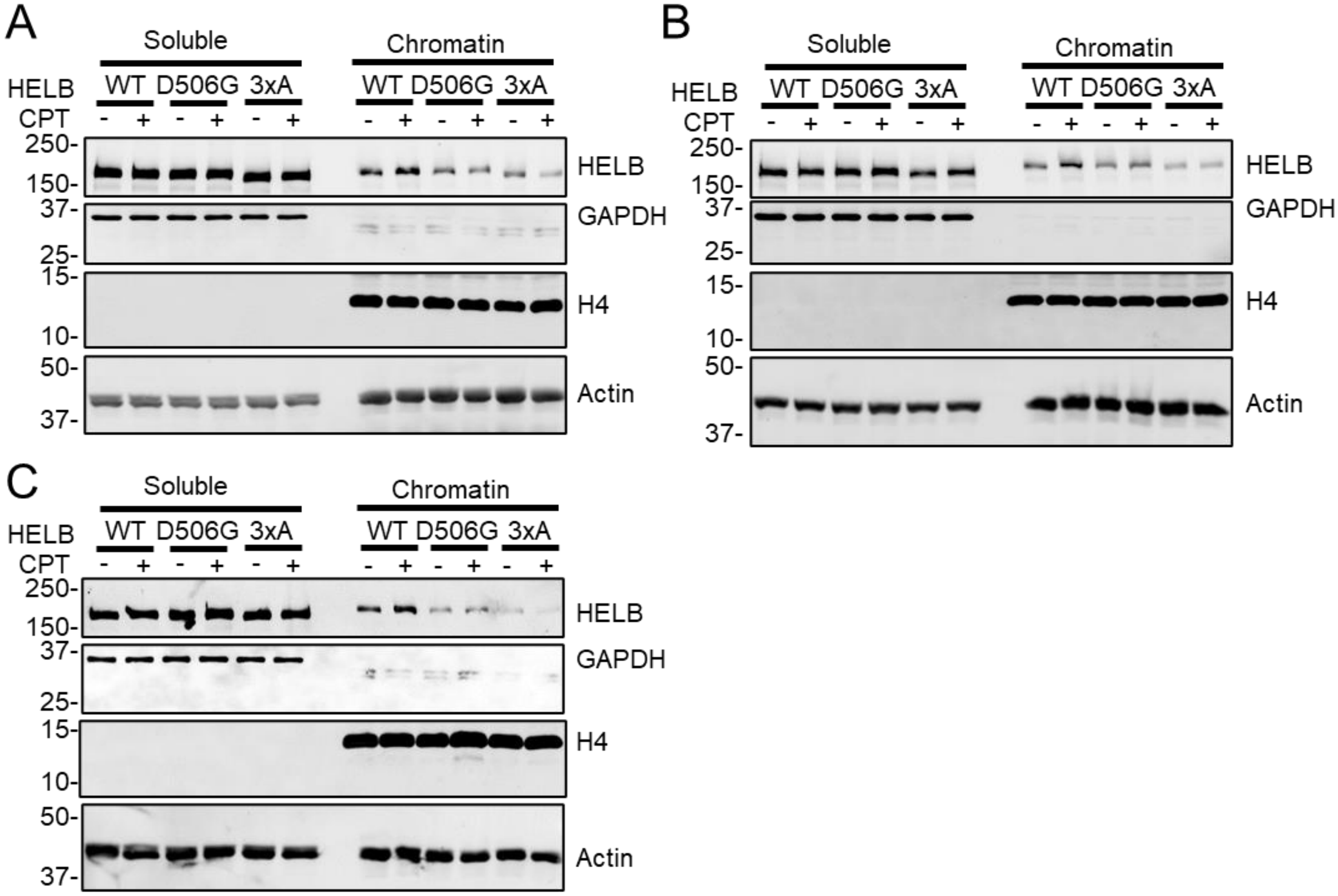

Supplementary Figure S7. D506G substitution in HELB interferes with localization of HELB to chromatin in response to replication stress. (**A-C**) Additional replicates of chromatin fractionation shown in Figure 4C-D. Lysates of $HELB^{KO}$ HEK293T cells expressing SFB-HELB variants (WT, D506G, and 3xA) with or without CPT treatment were separated into soluble and chromatin fractions and visualized by western blotting.

Supplementary Video V1. 60% of WT GFP-HELB co-localizes with mCherry-labeled DSBs. Shown is a 3D reconstruction of z-stacks of U2OS DSB reporter cells expressing GFP-HELB (WT) treated with 1 µM 4-OHT and 1 µM Shield1 before fixing and staining with an antibody to mCherry.

Supplementary Video V2. 40% of WT GFP-HELB does not co-localize with mCherry-labeled DSBs. Shown is a 3D reconstruction of z-stacks of U2OS DSB reporter cells expressing GFP-HELB (WT) treated with 1 µM 4-OHT and 1 µM Shield1 before fixing and staining with an antibody to mCherry.

References

1. Goddard,T.D., Huang,C.C., Meng,E.C., Pettersen,E.F., Couch,G.S., Morris,J.H. and Ferrin,T.E. (2018) UCSF ChimeraX: Meeting modern challenges in visualization and analysis. *Protein Sci*, **27**, 14–25.

2. Pettersen,E.F., Goddard,T.D., Huang,C.C., Meng,E.C., Couch,G.S., Croll,T.I., Morris,J.H. and Ferrin,T.E. (2021) UCSF ChimeraX: Structure visualization for researchers, educators, and developers. *Protein Sci*, **30**, 70–82.

3. Meng,E.C., Goddard,T.D., Pettersen,E.F., Couch,G.S., Pearson,Z.J., Morris,J.H. and Ferrin,T.E. (2023) UCSF ChimeraX: Tools for structure building and analysis. *Protein Sci*, **32**, e4792.
